# bZIP transcription factors PcYap1 and PcRsmA link oxidative stress response to secondary metabolism and development in *Penicillium chrysogenum*

**DOI:** 10.1186/s12934-022-01765-w

**Published:** 2022-04-02

**Authors:** W. D. Pérez-Pérez, U. Carrasco-Navarro, C. García‑Estrada, K. Kosalková, M. C. Gutiérrez-Ruíz, J. Barrios-González, F. Fierro

**Affiliations:** 1 Departamento de Biotecnología, Universidad Autónoma Metropolitana Unidad Iztapalapa, Iztapalapa, Ciudad de México, México; 2 INBIOTEC, Instituto de Biotecnología de León, Parque Científico de León, León, Spain; 3 Área de Medicina Experimental Y Traslacional, Departamento de Ciencias de La Salud, Universidad Autónoma Metropolitana Unidad Iztapalapa, Iztapalapa, Ciudad de México, México; 4 Laboratorio de Medicina Experimental, Unidad de Medicina Traslacional, IIB, UNAM/Instituto Nacional de Cardiología Ignacio Chávez, Tlalpan, Ciudad de México, México

**Keywords:** bZIP transcription factor, Yap1, RsmA, DNA-binding proteins, Transcriptional regulation, Secondary metabolism, Fungal morphogenesis, Reactive oxygen species, Oxidative stress defense

## Abstract

**Background:**

Reactive oxygen species (ROS) trigger different morphogenic processes in filamentous fungi and have been shown to play a role in the regulation of the biosynthesis of some secondary metabolites. Some bZIP transcription factors, such as Yap1, AtfA and AtfB, mediate resistance to oxidative stress and have a role in secondary metabolism regulation. In this work we aimed to get insight into the molecular basis of this regulation in the industrially important fungus *Penicillium chrysogenum* through the characterization of the role played by two effectors that mediate the oxidative stress response in development and secondary metabolism.

**Results:**

In *P. chrysogenum*, penicillin biosynthesis and conidiation are stimulated by the addition of H_2_O_2_ to the culture medium, and this effect is mediated by the bZIP transcription factors PcYap1 and PcRsmA. Silencing of expression of both proteins by RNAi resulted in similar phenotypes, characterized by increased levels of ROS in the cell, reduced conidiation, higher sensitivity of conidia to H_2_O_2_ and a decrease in penicillin production. Both PcYap1 and PcRsmA are able to sense H_2_O_2_-generated ROS in vitro and change its conformation in response to this stimulus. PcYap1 and PcRsmA positively regulate the expression of *brlA*, the first gene of the conidiation central regulatory pathway. PcYap1 binds in vitro to a previously identified regulatory sequence in the promoter of the penicillin gene *pcbAB*: TTAGTAA, and to a TTACTAA sequence in the promoter of the *brlA* gene, whereas PcRsmA binds to the sequences TGAGACA and TTACGTAA (CRE motif) in the promoters of the *pcbAB* and *penDE* genes, respectively.

**Conclusions:**

bZIP transcription factors PcYap1 and PcRsmA respond to the presence of H_2_O_2_-generated ROS and regulate oxidative stress response in the cell. Both proteins mediate ROS regulation of penicillin biosynthesis and conidiation by binding to specific regulatory elements in the promoters of key genes. PcYap1 is identified as the previously proposed transcription factor PTA1 (Penicillin Transcriptional Activator 1), which binds to the regulatory sequence TTAGTAA in the *pcbAB* gene promoter. This is the first report of a Yap1 protein directly regulating transcription of a secondary metabolism gene. A model describing the regulatory network mediated by PcYap1 and PcRsmA is proposed.

**Supplementary Information:**

The online version contains supplementary material available at 10.1186/s12934-022-01765-w.

## Background

In filamentous fungi, reactive oxygen species (ROS) have been shown to trigger and/or modulate different morphogenic processes [1–4] as well as fungus-plant interactions and biocontrol of plant pathogens [5–7]. A role of ROS in fungal secondary metabolism is currently well established too [8, 9]. The biosynthesis of aflatoxins by several *Aspergillus* species is the best-known case of induction of secondary metabolite (SM) biosynthesis by oxidative stress ([10] and references therein). Similarly, the production of trichothecene and expression of *Tri* genes in *Fusarium graminearum* is stimulated by treatment with H_2_O_2_ [11], and the production of lovastatin by *Aspergillus terreus* is modulated by ROS levels, which also regulate the expression of *lovE*, the lovastatin cluster regulatory gene [12]. It has been proposed that the biosynthesis of aflatoxin and trichothecene are part of the defense response against oxidative stress [13, 14].

The response to stress conditions in filamentous fungi shares common features with that in yeasts. A multistep phosphorelay system module transduces the signal to the stress-activated protein kinase/mitogen-activated protein kinase (SAPK/MAPK) module, which results in the activation of specific transcription factors that regulate the expression of target genes involved in the cellular response to the stress signals [8, 15–20]. Some of these transcription factors, such as AP-1, AtfA, AtfB, and MsnA, have been shown to participate directly or indirectly in the regulation of SM biosynthesis ([8, 21–24] and references therein).

*Penicillium chrysogenum* is one of the most important microorganisms in the biotechnological industry as a producer of penicillin and other β-lactam antibiotic precursors [25, 26]. Penicillin is synthesized from three precursor amino acids (α-aminoadipate, cysteine and valine) in three steps catalyzed by the enzymes δ(α-aminoadipyl)-cysteinyl-valine (ACV) synthetase, isopenicillin N synthase and isopenicillin N acyltransferase, encoded by the *pcbAB*, *pcbC* and *penDE* genes, respectively [27]. The three genes form a cluster, with the *pcbAB* and *pcbC* genes being expressed in opposite orientation from promoters situated in a common intergenic region. Unlike many other fungal SM gene clusters, no specific transcription factor is present in the cluster to control the simultaneous expression of the three genes. Instead, several wide domain transcription factors regulate the timing and expression levels of the genes in response to a variety of nutritional and physiological cues [28]. Penicillin biosynthesis is a good example of a process subject to complex global regulatory networks and serves as a model to study fungal secondary metabolism regulation [29, 30].

Several wide domain transcription factors and cis-acting regulatory elements that control the expression of the penicillin genes have been identified (reviewed in [30]). In a previous work, a fragment-deletion screen of the *pcbAB* gene promoter fused to the *lacZ* reporter gene identified a region that was important for transcription [31]. Using electrophoretic mobility shift assay (EMSA) and uracil interference assay (UIA) the sequence TTAGTAA, located 766–760 bp upstream of the *pcbAB* ATG start codon, was shown to strongly bind an as-then-unidentified transcription factor, which was named PTA1 (Penicillin Transcriptional Activator 1). Deletion and mutations of this sequence confirmed its in vivo functionality in the transcriptional regulation of the *pcbAB* gene [31]. The TTAGTAA regulatory sequence shows a nucleotide change (TTCGTAA) in the promoter of *Penicillium nalgiovense*, another penicillin producer usually growing on ripened meat products [32]. The level of transcription of the *pcbC* gene in *P. nalgiovense* is much lower than that in *P. chrysogenum*. Partially purified protein extracts from both fungi failed to bind the TTCGTAA sequence, whereas the *P. chrysogenum* extract, but not the *P. nalgiovense* extract, strongly bound the TTAGTAA sequence [33]. These findings confirm the role of the TTAGTAA sequence as an important regulatory element for the transcription of the *pcbAB* gene and, possibly, the *pcbC* gene in *P. chrysogenum*.

The PTA1 binding sequence has structural similarity to sequences bound by the bZIP-type family of transcription factors AP-1 [34]. The AP-1-like factor Pap1 from the fission yeast has been shown to specifically recognize the sequences TTACGTAA and TTAGTAA [35]. In *Saccharomyces cerevisiae*, a family of eight transcription factors, the Yap family (Yap1-8), has been described as related to the AP-1 family [36]. In many respects, the Yap proteins are functionally redundant, activating the transcription of genes involved in stress response. However, they differ in their specific response to stress agents and also show differences in the discrimination of individual base changes in the DNA-binding sequence [36, 37]. The preferred target sequence of YAP1-4 is TTACTAA.

In filamentous fungi, putative homologs of Yap1 and Yap3 have been cloned and studied. Yap1-like proteins have been found in *Aspergillus fumigatus* (showing 59% sequence similarity to Yap1) [38] and other fungi (reviewed in [9] and [39]), and they have been shown to participate in the response to oxidative stress. In the case of *Aspergillus parasiticus* [21], *Aspergillus ochraceus* [40] and *F. graminearum* [41], Yap1-like transcription factors were also shown to have a role in the production of the secondary metabolites aflatoxin, ochratoxin A and trichothecene, respectively. For their part, using a multicopy-suppressor approach, Shaaban et al. [42] cloned the *rsmA* gene, which was able to restore the deficiency in SM production caused by the absence of the Velvet complex in an *A. nidulans* mutant strain. The *rsmA* gene encodes a bZIP transcription factor that shares 31% identity with *Candida albicans* FCR3 [43] and whose highest identity in the genome of *S. cerevisiae* is with the FCR3 homolog Yap3 [44]. RsmA binds in vitro to two sequences in the promoter of the sterigmatocystin cluster regulatory gene *aflR*: TTAGTAA (a typical Yap1-binding sequence) and TGACACA [44]. RsmA orthologs have been found in other fungi and shown to activate SM production [45, 46]. RsmA proteins do not exhibit a consistent pattern regarding the stress response, showing varying effects in different fungi concerning stress challenges [44–47].

Another bZIP fungal transcription factor, AtfB, has been described as mediating both oxidative stress response and secondary metabolism in some *Aspergillus* species [13, 23, 48]. AtfB was first identified in *Aspergillus oryzae* as an ATF/CREB family transcription factor involved in oxidative stress response and conidia tolerance to H_2_O_2_ [49]. Using a ChIP approach, Roze et al. [23] found that, in *A. parasiticus*, AtfB binds to the promoters of seven aflatoxin genes carrying CRE (cAMP Response Element) motifs (TKACGTMA), whereas EMSA revealed that AtfB binds to a probe containing a CRE-like (TGACATAA) and an AP-1 (TGAGTAC) site from the promoter of the aflatoxin gene *nor-1*. These sequences show also a resemblance to the TTAGTAA regulatory sequence present in the *P. chrysogenum pcbAB* gene promoter.

Taking together all these data, we hypothesized that penicillin biosynthesis is regulated by ROS and that the PTA1-binding site TTAGTAA plays a role in this process. Here we describe that the Yap1 ortholog of *P. chrysogenum*, PcYap1, binds to the TTAGTAA regulatory element in the *pcbAB* promoter and to a TTACTAA sequence in the promoter of the conidiation regulatory gene *brlA*. PcYap1 regulates penicillin biosynthesis, conidiation and participates in the oxidative stress response. We also show that PcRsmA regulates penicillin biosynthesis, binds to a sequence (TGAGACA) located 68 bp upstream of the PcYap1-binding site in the *pcbAB* gene promoter and to a CRE site (TTACGTAA) located in the *penDE* gene promoter, and plays similar roles to PcYap1 in conidiation and response to oxidative stress. The direct transcriptional activation by a Yap1 protein of an SM gene and the conidiation regulatory gene *brlA* by binding to their promoters had not been previously reported.

## Results

### Oxidative stress regulates penicillin biosynthesis

**Fig. 1:**
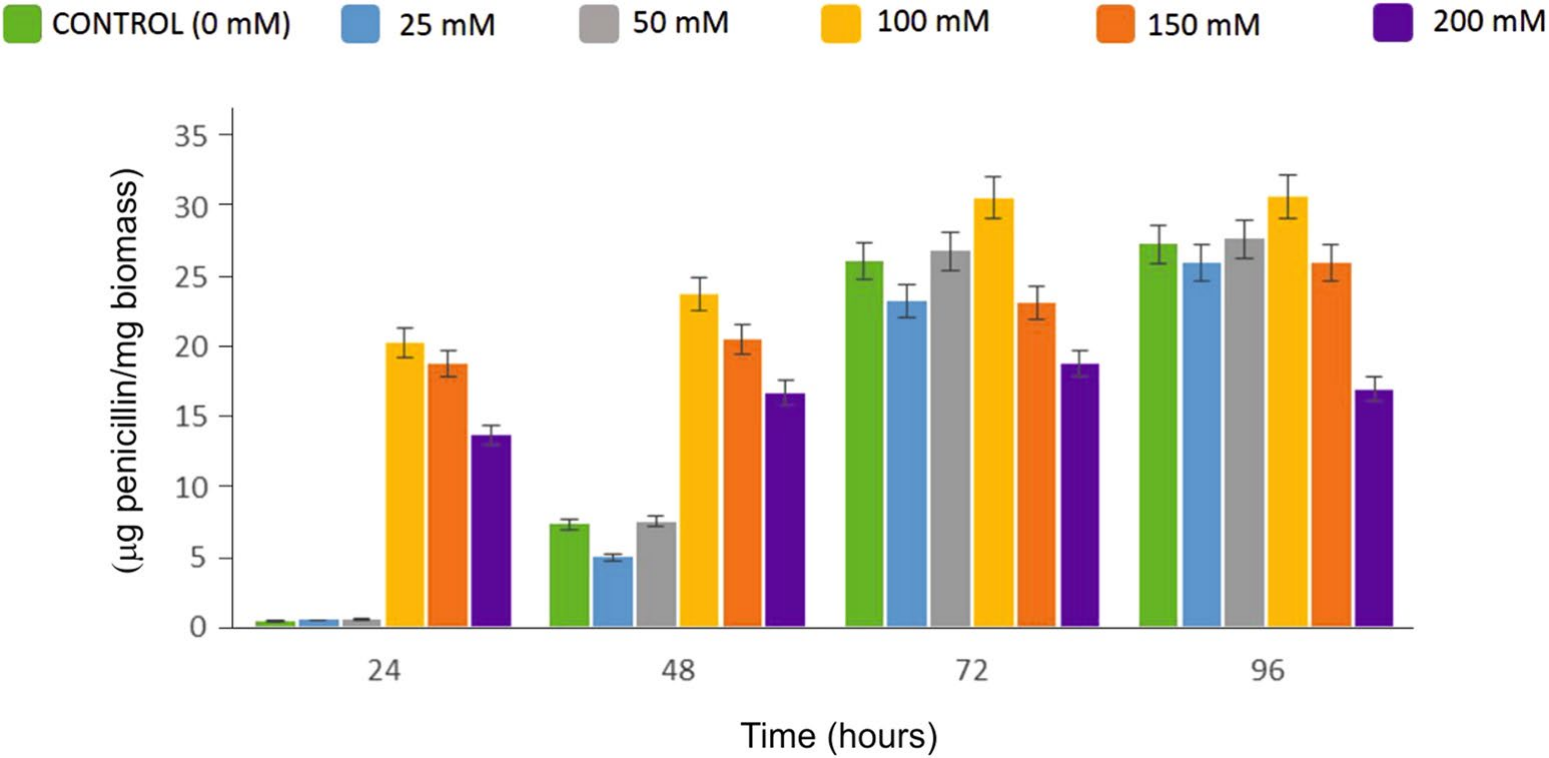
Penicillin production of strain *P. chrysogenum* Wis54-1255 in cultures submitted to oxidative stress. Different concentrations of H_2_O_2_ were added to the MCFP medium at the beginning of the cultivation time. Cultures were performed in triplicate, error bars represent standard deviation

Cultures of the *P. chrysogenum* Wis54-1255 strain were performed in flasks with complex production medium supplemented with H_2_O_2_ at concentrations of 25, 50, 100, 150 and 200 mM (Fig. 1). Concentrations up to 150 mM did not have any effect on the growth and biomass of the fungus, whereas 200 mM negatively affected growth. H_2_O_2_ concentrations of 25 and 50 mM did not have any significant effect on penicillin production, while concentrations of 100 mM and above produced important changes in the production pattern. Penicillin production started earlier when 100–200 mM H_2_O_2_ was added to the culture. With 100 mM H_2_O_2_, production was significantly higher until 72 h of cultivation, while 150–200 mM stimulated penicillin production only during the first 48 h. We concluded that a certain amount of H_2_O_2_-induced ROS positively regulates penicillin biosynthesis, and chose the concentration showing higher induction, 100 mM H_2_O_2_, for subsequent experiments.

### Yap1 and RsmA orthologs in the* P. chrysogenum* genome

A transcription factor homologous to Yap1 was the first candidate to bind to the TTAGTTA regulatory sequence in the *pcbAB* promoter. In a search of the *P. chrysogenum* Wis54-1255 (aka *P. rubens* Wis54-1255, taxid 500,485) genome at NCBI, we found the gene Pc20g15280, whose deduced amino acid sequence showed 28% overall identity with the Yap1 protein from *S. cerevisiae* (41% and 52% in two conserved regions of 107 and 58 amino acids located at the N- and C-terminal ends, respectively) and 63.6% overall identity with AfYap1 from *A. fumigatus*. No other protein with high similarity to Yap1 is encoded in the *P. chrysogenum* genome. Therefore, we considered Pc20g15280 a *Yap1* ortholog and named it *Pc-yap1*.

Another candidate to bind to the TTAGTAA sequence and regulate penicillin biosynthesis was RsmA, a Yap3-like protein that regulates secondary metabolism in *A. nidulans*, *A. fumigatus* and *Pestalotiopsis fici* (see “Background” section). The protein encoded by the Pc12g16510 gene in the *P. chrysogenum* genome shows 59.4% overall identity with RsmA from *A. nidulans* at the amino acid level, 43.2% identity with PfZipA (RsmA ortholog) from *P. fici*, and the highest similarity in the *S. cerevisiae* genome is with Yap3p (23.4% identity) and in *C. albicans* with Fcr3p (29.1% identity). Therefore, we named Pc12g16510 as *Pc-rsmA*. *Pc-yap1* and *Pc-rsmA* encode putative bZIP transcription factors with a deduced sequence of 582 (PcYap1) and 299 (PcRsmA) amino acids, which conserve the basic DNA-binding and dimerization leucine zipper domains typical of these proteins. We decided to characterize PcYap1 and PcRsmA function by two approaches: (1) EMSA analysis of the binding of the proteins to the TTAGTAA sequence and other regions in the penicillin gene promoters, and (2) Knocking down and overexpression of the genes, and characterization of the resulting phenotypes in relation to penicillin production, oxidative stress defense and conidiation.

### PcYap1, but not PcRsmA, binds to the* pcbAB* gene regulatory sequence TTAGTAA

**Fig. 2:**
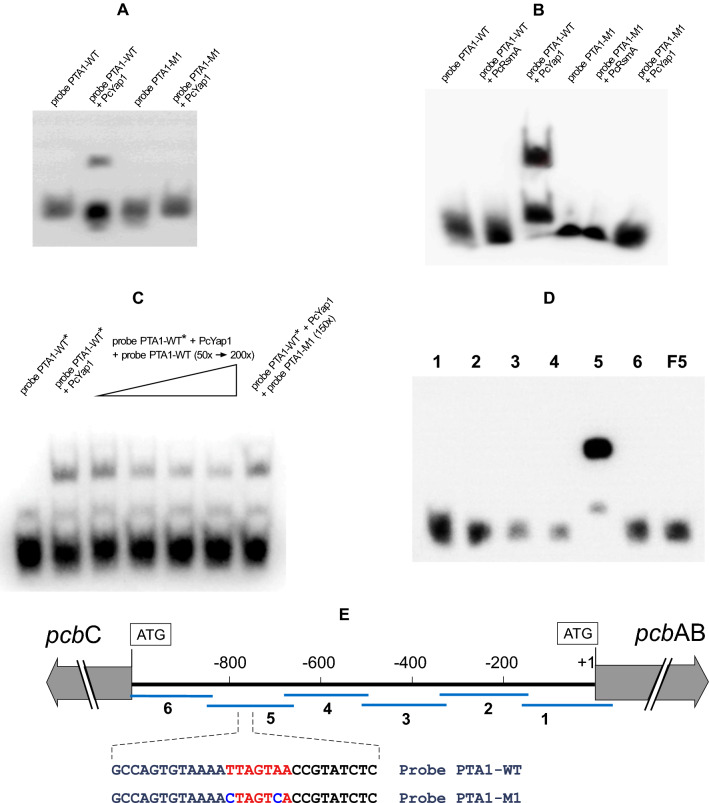
EMSA of PcYap1 binding activity in the *pcbAB-pcbC* intergenic region. **A** Positive binding reaction of PcYap1 with the probe PTA1-WT containing the regulatory sequence TTAGTAA, and lack of binding reaction with the probe PTA1-M1 containing the mutant sequence CTAGTCA. **B** Binding reactions of PcYap1 and PcRsmA with the probes PTA1-WT and PTA1-M1. **C** Specificity test for the binding of PcYap1 to the TTAGTAA sequence. PTA-WT* is the labelled probe; PTA-WT and PTA-M1 are cold (unlabelled) probes, which were added in excess amounts of 50×, 100×, 150× and 200× as indicated at the top of the gel image. **D** Screening of the entire *pcbAB-pcbC* intergenic region to locate target sites of PcYap1; lanes 1 through 6 correspond to binding reactions between probes 1 through 6 with PcYap1, lane F5 is the free probe 5. **E** Location in the *pcbAB* gene promoter of the six probes used in **D**; below is indicated the position and the sequence of the upper strand of probes PTA1-WT and PTA1-M1

Purified PcYap1 and PcRsmA (see “Materials and methods” section) were incubated with a 28-bp DNA probe from the *pcbAB* gene promoter containing the TTAGTAA sequence (probe PTA1-WT) and with a mutated probe with two base changes (probe PTA1-M1), and the reactions were run in a native PAGE (Fig. 2). The results showed that PcYap1 binds to the probe with the TTAGTAA sequence but not to the mutated probe, whereas PcRsmA does not bind to any of the probes. Next, we performed a specificity EMSA adding excess amounts of unlabelled PTA1-WT and PTA1-M1 probes to the binding reactions between PcYap1 and the PTA1-WT probe (Fig. 2C). Only the PTA1-WT probe competed with the labelled probe for binding to PcYap1, thus demonstrating that the binding of PcYap1 to the TTAGTAA sequence is specific.

We then searched for other possible PcYap1 binding sites by performing EMSA with six probes covering the entire *pcbAB-pcbC* intergenic region. As shown in Fig. 2D, the only probe interacting with PcYap1 was probe 5, which contains the TTAGTAA sequence already proven to be bound by the protein. Therefore, PcYap1 has only one binding site in the *pcbAB-pcbC* intergenic region.

### Both PcYap1 and PcRsmA regulate penicillin biosynthesis

**Fig. 3:**
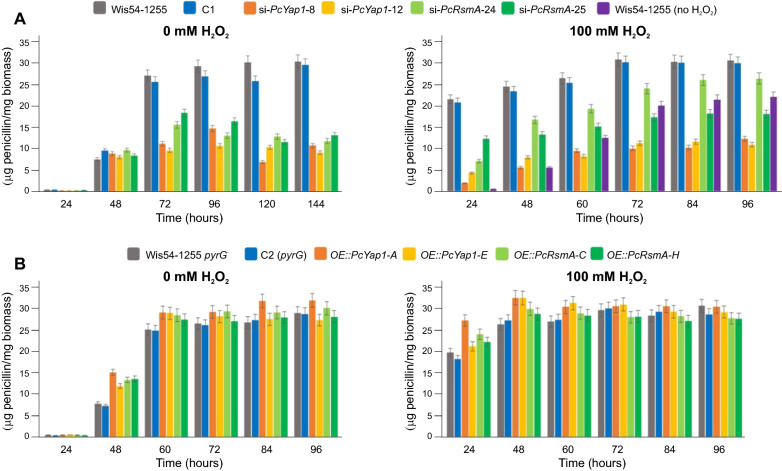
Penicillin production in strains with knocked down expression of *Pc-yap1* and *Pc-rsmA* (**A**) and strains overexpressing the respective genes (**B**); strain C1 contains the empty pGpdPki-RNAi vector used for gene silencing and strain C2 (*pyrG*) contains the empty pBKSpyrG vector used for gene overexpression. To test the effect of H_2_O_2_-generated oxidative stress, 100 mM H_2_O_2_ was added at the beginning of the cultivation time. In the panel at the right in **A** the extra bar (violet color) corresponds to the parental strain Wis54-1255 grown on medium without added H_2_O_2_. Bar sizes are the result of three biological replicas, error bars correspond to standard deviation. See “Materials and methods” section for details

To test the possible involvement of PcYap1 and PcRsmA in the regulation of penicillin production, we carried out cultures in flasks with two *Pc-yap1*-knocked down strains (si-*PcYap1*-8 and -12) and two *Pc-rsmA*-knocked down strains (si-*PcRsmA*-24 and -25). In CP medium without H_2_O_2_, the behavior of the strains with knocked down *Pc-yap1* and *Pc-rsmA* expression was very similar, showing a clear reduction in penicillin production with respect to the control strains from 72 h of cultivation onwards, accumulating at the end of the cultures around 33% and 41% of the amount in the control strains, respectively (Fig. 3A). When 100 mM H_2_O_2_ was added to the culture, penicillin production started earlier in all strains, and differences in production showed up from 24 h of cultivation (Fig. 3B). In this case, the effect of *Pc-rsmA* silencing was less marked than that observed in cultures without H_2_O_2_, with penicillin titers of 60–87% compared to the values obtained with the control strains. The final amount of penicillin produced by strain Wis54-1255 when was submitted to oxidative stress was 1.38-fold higher than in the absence of H_2_O_2_ (purple bars); this H_2_O_2_-mediated induction did not take place when expression of *Pc-yap1* was knocked down, and in fact, production in *PcYap1*-knocked down strains submitted to oxidative stress was only 53% of that in strain Wis54-1255 not submitted to stress. For its part, knocking down of *Pc-rsmA* also abolished the inducing effect of H_2_O_2_ in the late hours of the culture, but in this case penicillin production was similar to that in strain Wis54-1255 not submitted to stress.

When the *Pc-yap1* and *Pc-rsmA* genes were overexpressed under the control of the constitutive *pki* promoter, a 1.8-fold increase in penicillin production was observed at 48 h of cultivation in medium without H_2_O_2_ in both cases (Fig. 3C), whereas in cultures supplemented with 100 mM H_2_O_2_ differences between *OE* and control strains were significant only in *OE::PcYap1* strains at 48 h (1.22-fold) (Fig. 3D).

From these results, we concluded that both PcYap1 and PcRsmA are important positive regulators of penicillin biosynthesis and that the induction of penicillin production by H_2_O_2_-generated oxidative stress is mediated by PcYap1 and, to a lesser extent, by PcRsmA.

### PcRsmA binds specifically to a TGAGACA sequence in the* pcbAB-pcbC* intergenic region

**Fig. 4:**
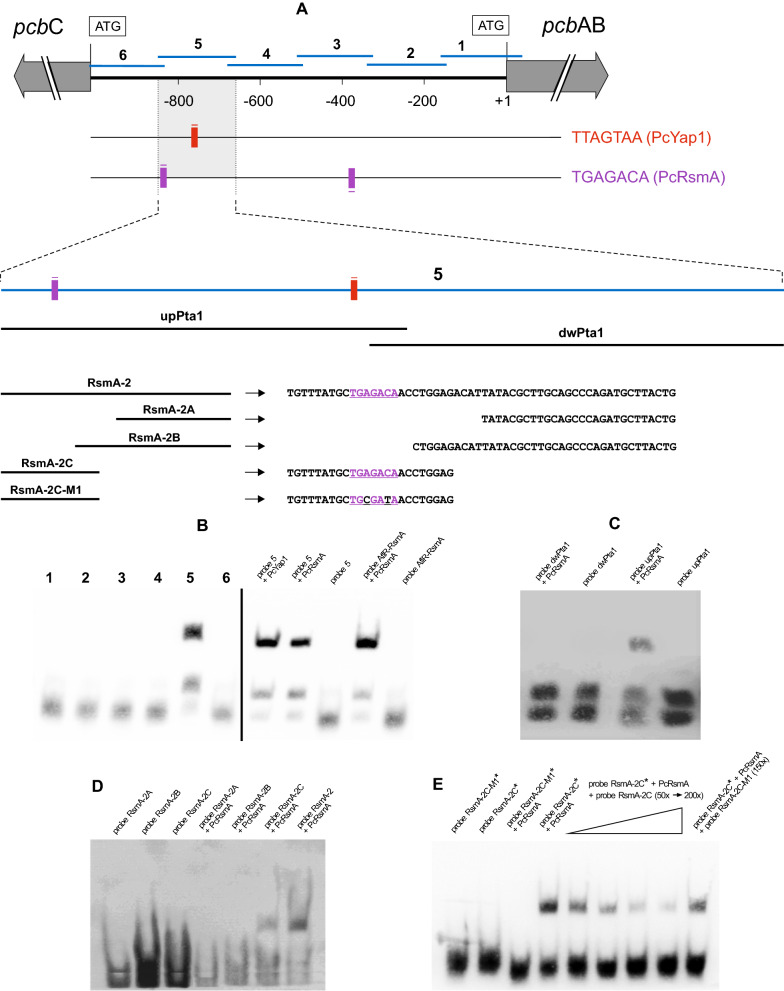
EMSA of the PcRsmA binding activity in the *pcbAB-pcbC* intergenic region. **A** The *pcbAB-pcbC* intergenic region is shown at the top, with the position of the six probes used for screening the DNA binding activity of PcYap1 (Fig. 2D) and PcRsmA. The position of the DNA binding sites of PcYap1 and PcRsmA is shown with a bar of red and violet color, respectively. The small lines above or below the bars indicate the position of the binding sequence in the upper or lower strand, respectively. Below it is shown the relative position of seven probes located within the DNA region corresponding to probe 5, with the sequence of the upper strand from the smaller probes at their right. **B** Screening of the entire *pcbAB-pcbC* intergenic region to locate target sites of PcRsmA; lanes 1 through 6 correspond to binding reactions between probes 1 through 6 with PcRsmA. At the right are shown confirmation binding reactions of PcYap1 and PcRsmA with probe 5, and a positive control reaction of PcRsmA with probe AflR-RsmA of 25 bp (Additional file 12), which belongs to a region in the promoter of the *A. nidulans* sterigmatocystin cluster gene *aflR* containing a TGACACA sequence proven to be bound by *A. nidulans* RsmA [44]. **C** Positive binding reaction of PcRsmA with the probe upPta1 and negative reaction with the probe dwPta1. **D** Location of the PcRsmA binding sequence; the probes containing the sequence TGAGACA (RsmA-2 and RsmA-2C) are bound by PcRsmA, whereas the probes lacking this sequence (RsmA-2A and RsmA-2B) are not. **E** Specificity test for the binding of PcRsmA to the TGAGACA sequence. We used the probe RsmA-2C-M1, containing the mutated sequence TGCGATA, to prove the specificity. RsmA-2C* and RsmA-2C-M1* are labelled probes; RsmA-2C and RsmA-2C-M1 are cold (unlabelled) probes, which were added in excess amounts of 50x, 100x, 150× and 200× as indicated at the top of the gel image

As concluded in the previous sections, PcRsmA is a positive regulator of penicillin biosynthesis but does not bind to the TTAGTAA regulatory sequence (Fig. 2B). In an attempt to identify possible target sites of PcRsmA we performed EMSAs using six probes of around 190 bp each covering the entire *pcbAB-pcbC* intergenic region (Fig. 4A). The results showed that only probe 5 was bound by PcRsmA, the same probe in which the PcYap1-binding sequence is located (Fig. 4B). Next, we divided probe 5 into two halves (probes upPta1 and dwPta1), and PcRsmA bound only to upPta1, which comprises the DNA region around and upstream of the PcYap1-binding sequence (Fig. 4C). Within this region and 68 bp upstream of TTAGTAA, there is a sequence (TGAGACA) that shows similarity to a TGACACA sequence present in the *A. nidulans aflR* gene promoter, previously proven to be bound by RsmA, and to putative RsmA-binding sequences found in a MEME analysis of promoters of genes up-regulated in an *A. nidulans OE::rsmA* strain [44]. Then, we designed several probes containing and excluding the TGAGACA sequence, and found that PcRsmA only bound to those containing this sequence (Fig. 4D). Specificity of the binding of PcRsmA to the TGAGACA sequence was confirmed by mutating the sequence at positions 3 and 6 to TGCGATA (probe RsmA-2C-M1) and by adding excess amounts of non-labelled probes in competition assays (Fig. 4E). Two TGAGACA sequences are present in the *pcbAB-pcbC* intergenic region: the PcRsmA-bound sequence at position -835 from the *pcbAB* gene start codon (named TGAGACA-2), and an unbound sequence at position -377 in reverse orientation (named TGAGACA-1), which is present in probe 3 (Fig. 4A). The fact that only one of the two TGAGACA sequences in the *pcbAB-pcbC* intergenic region is bound by PcRsmA indicates that its binding to this sequence is context-dependent, and additional nucleotides must be required for it.

The proximity of the PcYap1-binding site (TTAGTAA) to the PcRsmA binding site (TGAGACA) prompted us to explore the possibility that both proteins interact when binding to their respective target sites. We designed an EMSA with several probes of this region that contained one or the other binding site, or both, incubating them with PcYap1, PcRsmA, or both proteins together, expecting to get a supershift in the case that the proteins interact (Additional file 7). Only when the probe contained both binding sites (probe upPta1) and was incubated with both proteins together a supershift pattern appeared, probably as a result of the simultaneous binding of each protein to its specific binding site. This indicates that apparently there is no in vitro interaction between the proteins when binding to their respective target sites.

### PcRsmA, but not PcYap1 or PcAtf21, binds to a CRE motif (TKACGTMA) in the* penDE* gene promoter

Another transcription factor involved in both oxidative stress response and secondary metabolism described in some *Aspergilli* is AtfB/Atf21 [8, 48], which belongs to the ATF/CREB family of transcription factors. Hai and Curran (1991) [50] reported that these factors bind as homo or heterodimers to the consensus sequence TKACGTMA. This sequence matches the TTAGTAA regulatory sequence in the *pcbAB-pcbC* intergenic region but for the presence of an additional S base, and one such sequence (TTACGTAA) is present in the promoter region of the *penDE* gene at position -697 from the ATG start codon. Therefore, we decided to investigate if a *P. chrysogenum* AtfB homologue was able to bind some of these motifs. A Blast search in the *P. chrysogenum* genome using the sequence of *Aspergillus flavus* Atf21 (XP_002381221) as bait identified a protein of 319 amino acids encoded at locus Pc21g08330, which showed 45.8% overall identity to *A. flavus* Atf21, *A. parasiticus* AtfB and *A. oryzae* AtfB. The highest identity of Pc21g08330 was to *Aspergillus clavatus* Atf21 (58.7%) and *A. fumigatus* Atf21 (54.9%), and the highest identity in the *Schizosaccharomyces pombe* genome was to the ATF-CREB family protein Atf21 (NP_595707), 9.1% identity head to tail, similar to that shown by different *Aspergillus* AtfB/Atf21 proteins, with 37% identity in a 135 amino acid region containing a bZIP-ATF2 motif. Pc21g08330 is different from a putative Atf1/AtfA ortholog (locus Pc13g09580) and other putative ATF/CREB proteins in the *P. chrysogenum* genome. Therefore, we decided to name the protein encoded at locus Pc21g08330 as PcAtf21.

**Fig. 5:**
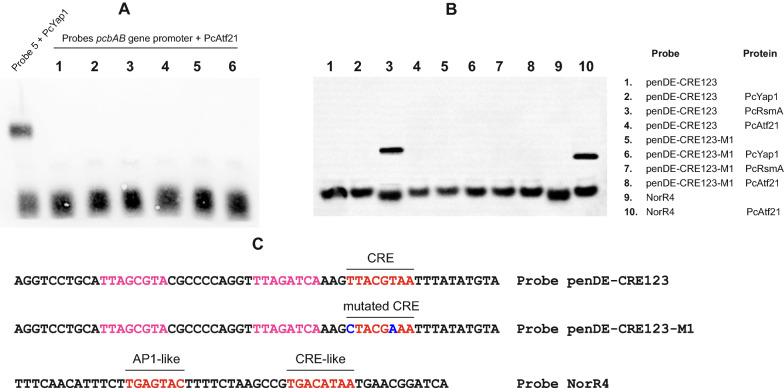
EMSA of the PcAtf21 binding activity in the *pcbAB-pcbC* intergenic region and of the three bZIP transcription factors in a region of the *penDE* gene promoter. **A** The six probes (lanes 1 to 6) of the *pcbAB-pcbC* intergenic region (Fig. 4A) were tested for binding to the PcAtf21 transcription factor; a binding reaction with probe 5 and PcYap1 was used as positive control. **B** EMSA of the PcYap1, PcRsmA, and PcAtf21 binding activities with probe penDE-CRE123, whose sequence corresponds to a central region in the *penDE* gene promoter; probe norR4 was used as positive control for PcAtf21 binding (see text). **C** Sequences of the probes used for EMSA. Probe penDE-CRE123 contains a CRE site (highlighted in red) and two CRE-like sites upstream (highlighted in pink); the nucleotide changes at the CRE site in the probe penDE-CRE123-M1 are highlighted in blue

PcAtf21 was expressed heterologously as a *c-myc*-6xHis-tagged protein in *Pichia pastoris*, purified and used for EMSA (see “Materials and methods” section). PcAtf21 did not bind to any of the six probes covering the *pcbAB-pcbC* intergenic region (Fig. 5A), which ruled out the possibility that it recognizes the regulatory TTAGTAA sequence located in probe 5. This result is in agreement with the absence of CRE motifs in the *pcbAB-pcbC* intergenic region. PcAtf21 functionality for in vitro binding was demonstrated by its capacity to bind the NorR4 probe (Fig. 5B, lane 10), whose sequence belongs to the *A. parasiticus nor-1* gene promoter and contains an AP-1-like (TGAGTAC) and a CRE-like site (TGACATAA) (see “Discussion” section).

Next, we tested if a 57-bp probe (penDE-CRE123), which belongs to the *penDE* gene promoter and contains the CRE site TTACGTAA plus two additional CRE-like sequences upstream of it, was bound by some of the three bZIP transcription factors under study. No binding could be detected with PcYap1 and PcAtf21, whereas PcRsmA bound to the probe producing a clear gel retardation pattern (Fig. 5B, lane 3). When the CRE site in the probe was mutated to CTACGAAA binding was abolished, which strongly suggests that PcRsmA specifically recognizes the TTACGTAA sequence. From these results, we concluded that PcAtf21 is not directly involved in the regulation of transcription of the penicillin genes, whereas PcRsmA probably regulates the expression of the *penDE* gene.

### Both PcYap1 and PcRsmA participate in oxidative stress defense

**Fig. 6:**
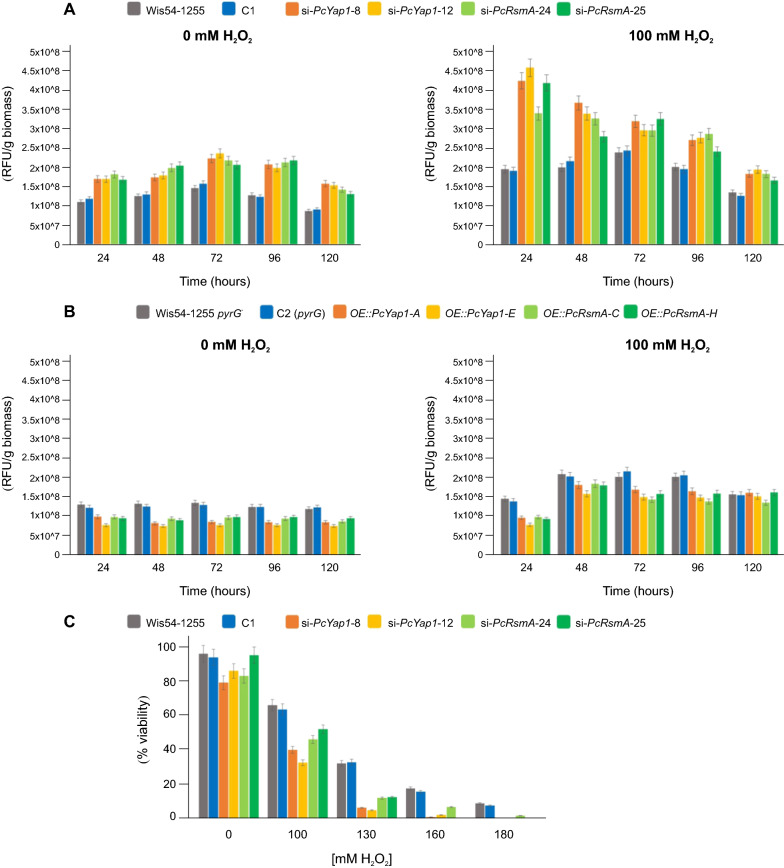
Effect of PcYap1 and PcRsmA on oxidative stress response. Effect of *Pc-yap1* and *Pc-rsmA* silencing (**A**) and overexpression (**B**) on the amount of naturally occurring and H_2_O_2_-generated ROS in mycelium from submerged cultures. Strain C1 contains the empty pGpdPki-RNAi vector used for gene silencing and strain C2 (*pyrG*) contains the empty pBKSpyrG vector used for gene overexpression. Results are expressed in relative fluorescence units (RFU) per gram of biomass (dry weight) (see “Materials and methods” section for details). **C** Effect of *Pc-yap1* and *Pc-rsmA* silencing on the viability of conidia submitted to oxidative stress with different concentrations of H_2_O_2_. Results are expressed as percentage of conidia able to form colonies on plates with respect to the initial number counted under the microscope (see “Materials and methods” section for details). In all cases bar sizes are the result of three biological replicas, error bars correspond to standard deviation

To test the possible involvement of PcYap1 and PcRsmA in the defense against ROS, we measured the amount of ROS present in cultures of knocked-down and overexpressing strains in MCFP medium with or without H_2_O_2_. The two proteins showed very similar profiles in the effect caused on the intracellular ROS levels (Fig. 6). In strains with RNAi-mediated attenuation of PcYap1 or PcRsmA expression, ROS levels were higher than in the control strains throughout the cultivation time (Fig. 6A). The highest difference occurred at 24 h in the presence of 100 mM H_2_O_2_, when the *Pc-yap1*-knocked down strains presented 2.28-fold higher amounts of ROS than the control strains and the *Pc-rsmA*-knocked down strains showed a 1.96-fold increase. In this condition, the differences in ROS levels between strains became smaller as the culture progressed, reflecting the impact of the addition of H_2_O_2_ at the beginning of the culture. In cultures without H_2_O_2_, differences between either *Pc-yap1*- or *Pc-rsmA*-knocked down strains and control strains oscillated in the range of 1.28-fold and 1.75-fold, with a tendency to increase as the culture progressed.

When either *Pc-yap1* or *Pc-rsmA* were overexpressed, the ROS levels showed a decrease with respect to those in the control strains (Fig. 6B). In the case of the strains overexpressing *Pc-yap1*, this effect was more noticeable in the cultures without H_2_O_2_ (ROS levels were 61% of those present in the control strains at 48–72 h), whereas in strains overexpressing *Pc-rsmA*, ROS levels were around 71% of those in the control strains at 48 h in cultures without H_2_O_2_ and oscillated between 68 and 72% in cultures with H_2_O_2_.

Participation of PcYap1 and PcRsmA in defense against ROS was further confirmed by assaying the viability of conidia at different concentrations of H_2_O_2_ and the effect that knocking-down of both genes had on viability. As shown in Fig. 6C, viability is increasingly affected by silencing of *Pc-yap1* and *Pc-rsmA* as conidia are exposed to higher concentrations of H_2_O_2_. With 130 mM H_2_O_2_, the number of viable conidia in *Pc-yap1*- and *Pc-rsmA*-knocked down strains was 17.3% and 37.6%, respectively, of that in control strains. In the *Pc-yap1*-knocked down strains, no viable conidia were found with 180 mM H_2_O_2_, whereas *Pc-rsmA*-knocked down strains showed differences between them in conidia viability at high H_2_O_2_ concentrations, with 0% viability in strain si-*PcRsmA*-25 at 160 and 180 mM.

### PcYap1 and PcRsmA respond to the presence of H_2_O_2_-generated ROS in vitro

**Fig. 7:**
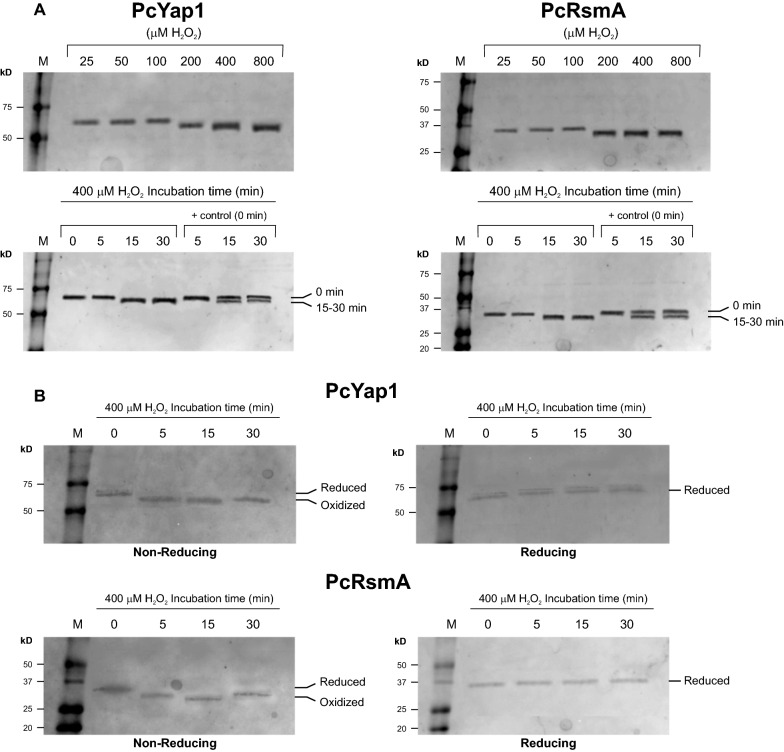
Mobility changes in SDS-PAGE of purified PcYap1 and PcRsmA incubated with H_2_O_2_ prior to loading on the gel. **A** An H_2_O_2_ concentration gradient was used to determine the amount of H_2_O_2_ producing changes in mobility (upper panels); incubation time was 15 min. In the lower panels, proteins were incubated with a concentration of 400 µM for different times; the three lanes at the right of each panel were loaded with a control (no incubation with H_2_O_2_) and the 400 µM H_2_O_2_-incubated protein simultaneously, with the purpose to showcase the different mobility of the bands corresponding to H_2_O_2_-treated and untreated proteins. Gels had an acrylamide concentration of 10%, the amount of protein used in each reaction was 25 µg. In all cases non-reducing conditions were used (see Materials and Methods and the text for details). **B** Comparison between reducing and non-reducing conditions in the mobility of the proteins after 0 to 30 min of incubation with 400 µM H_2_O_2_. Gels had an acrylamide concentration of 8%, the amount of protein used in each reaction was 15 µg

Once the participation of PcYap1 and PcRsmA in oxidative stress defense was confirmed, we analyzed wether they were affected in vitro by the presence of H_2_O_2_ to get insight into their possible role as ROS sensors. The purified PcYap1::*c-myc*-6xHis and PcRsmA::*c-myc*-6xHis proteins were incubated with increasing concentrations of H_2_O_2_ for 15 min and immediately loaded onto an SDS-PAGE gel under non-reducing conditions (see “Materials and methods” section). As shown in Fig. 7A, H_2_O_2_ concentrations of 200 µM and above produced a change in the mobility of both proteins, which run slightly faster in the gel. Then, the proteins were submitted to different incubation times with 400 µM H_2_O_2_, observing the same effect after 15 min of incubation. This change in mobility can be interpreted as a conformational change of the proteins, with the formation of disulphide bonds due to the oxidizing conditions produced by H_2_O_2_. In a work by Wood et al. [51], a fragment of *S. cerevisiae* Yap1 containing the two cysteine-rich domains fused to GFP was used to test the conformational changes the protein underwent in H_2_O_2_-treated cells with respect to untreated cells, and the results showed that the protein from H_2_O_2_-treated cells presented an oxidized form with disulphide bonds (as determined by NMR spectroscopy) that moved faster in SDS-PAGE gels in non-reducing conditions. The results in Fig. 7 are analogous to those of Wood et al. [51] and can thus be interpreted in the same way. To confirm that the higher mobility observed when the proteins were incubated with H_2_O_2_ was due to disulphide bond formation, we run the H_2_O_2_-incubated proteins in SDS-PAGE in reducing and non-reducing conditions (Fig. 7B). As expected, the mobility change observed in non-reducing conditions did not occur in reducing conditions, a result that can be explained by the rupture of the disulphide bonds upon reduction of the cysteine residues and the subsequent conformational change.

In *S. cerevisiae*, Yap1 moves into and out from the nucleus under normal physiological conditions. In oxidative conditions caused by H_2_O_2_, disulphide bonds form, and the bonding between C303 and C598 produces a conformational change that masks the Nuclear Export Signal (NES) located at the C-terminus, thus preventing recognition by the exportin Crm1 and export from the nucleus [51–53]. PcYap1 shows high identity with Yap1 and very high identity with filamentous fungi Yap1 orthologs, and contains all the conserved domains found in these organisms: Nuclear Localization Signal (NLS), NES, bZIP, CRD (cysteine rich domains) (Additional file 8). Nuclear accumulation of filamentous fungi Yap1 orthologs upon induction of oxidative stress conditions has been observed in all cases studied [39]. Besides, in a STRING analysis, PcYap1 showed interaction with a *P. chrysogenum* exportin Crm1 ortholog (Pc16g01720). Therefore, PcYap1 very likely follows an activation/nuclear localization pattern similar to those described for other fungi. Notwithstanding the results of Fig. 7 indicate that PcYap1 can directly sense oxidizing conditions, its activation in the cell may also be achieved through other mechanisms, such as Gpx3-mediated oxidation, as established for *S. cerevisiae* [53], or by other peroxiredoxins, like Asp f3 from *A. fumigatus* which is required for Afyap1 activation and nuclear localization [54].

PcRsmA shows the same capacity of ROS-sensing and conformational change in vitro as PcYap1. Analysis of the protein sequence reveals the presence of only two cysteine residues for the formation of disulphide bonds, located at the C-terminus (Additional file 9). The one at position 271 is conserved in *A. nidulans* RsmA and *S. cerevisiae* Yap3, and the one at position 228 only in RsmA. A putative NES is located between these two cysteine residues, and thus might be affected by the formation of a disulphide bond. Yap3 localizes in the nucleus upon treatment with hydroquinone [55]; however, whether the localization mechanism is similar to that of Yap1 remains to be elucidated. Therefore, with the currently available data, we cannot propose a specific mechanism for the entrance/location of PcRsmA in the nucleus.

### PcRsmA controls expression of the* Pc-yap1* gene

**Fig. 8:**
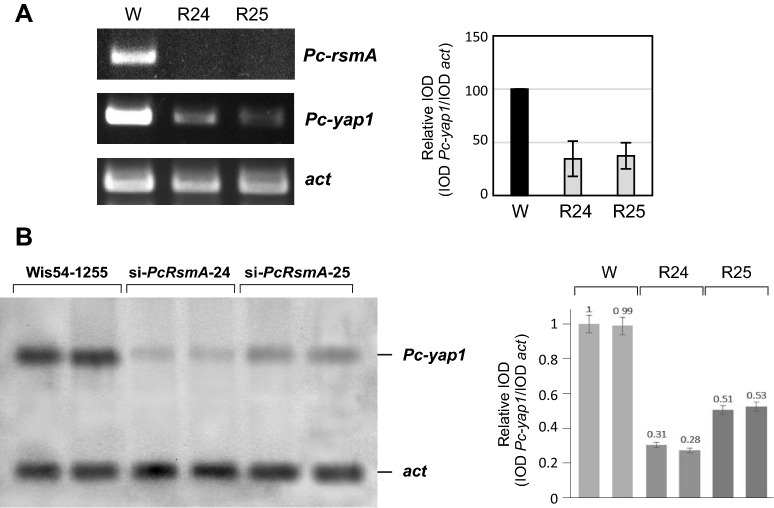
Regulation of the expression of the *Pc-yap1* gene by PcRsmA. **A** Semiquantitative RT-PCR using as template RNA extracted from mycelium grown for 36 h in submerged cultures (medium MPPY); primers were the same as indicated in the legend to Fig. 3. The left panel shows the intensity of the bands in an agarose gel loaded with the products of the RT-PCR reactions, and the right panel the densitometry analysis of the bands. The results were normalized with the bands of the constitutively expressed *act* gene, and the parental strain Wis54-1255 (lane W) was used as reference with a value for integrated optical density (IOD) of 100. R24: strain si-*PcRsmA*-24; R25: strain si-*PcRsmA*-25. **B** Northern blot with RNA extracted from mycelium grown on solid Power medium for 120 h; the probes used were a 458 bp fragment from the *Pc-yap1* gene amplified by PCR with primers siYAP1-F and -R, and a 508 bp fragment from the *act* gene amplified by PCR with primers N-actA-1F and R (Additional file 12). At the right, the densitometry analysis was performed as described above, adjusting the IOD of strain Wis54-1255 to 1. (See “Materials and methods” section for details)

The ability of the transcription factor PcRsmA to participate in the response to oxidative stress in the cell may be due to direct regulation of antioxidant enzyme-encoding genes and/or the regulation of wide domain factors involved in oxidative stress defense, such as PcYap1. We explored this latter possibility by measuring the levels of *Pc-yap1* transcript in *Pc-rsmA*-knocked down strains by RT-PCR (with RNA from mycelium grown in submerged cultures) and by Northern blot (with RNA from mycelium grown on solid medium) (Fig. 8). The results showed that in submerged cultures *Pc-yap1* expression in the *Pc-rsmA*-silenced strains was around 35% of that in the control strain, whereas in solid medium it was 29–52%. Therefore, PcRsmA positively regulates transcription of the *Pc-yap1* gene. This regulation may be direct, by binding of PcRsmA to the *Pc-yap1* gene promoter, or indirect. No putative PcRsmA binding sites with the sequences TGASACA or TKACGTMA are present in the promoter, but a typical AP-1 binding site, TGAGTCA, is present 145 bp upstream of the ATG start codon, although binding of PcRsmA to this sequence was not tested. The regulation of *Pc-yap1* gene expression by PcRsmA implies that this protein can regulate different processes in the cell directly by binding to the promoters of the corresponding genes, indirectly through upregulation of PcYap1 expression or in both ways, as happens in the case of penicillin biosynthesis, where both transcription factors have a direct role in the process.

### Conidiation is positively regulated by PcYap1 and by PcRsmA to a similar extent

Next, we studied the function of PcYap1 and PcRsmA in the growth and development of the fungus. First, we analyzed the radial growth of the colonies on plates with PDA medium, finding no significant differences between the strains with knocked down expression of the genes, the strains overexpressing the genes, and the control strains (Additional file 10). Biomass produced in submerged cultures was likewise not affected by PcYap1 or PcRsmA activity (data not shown). Therefore, neither of the proteins regulates the vegetative growth of the fungus. This result is in agreement with studies in other filamentous fungi, where inactivation or overexpression of Yap1 proteins usually has little to no effect on vegetative growth [39], with a few exceptions, such as *Alternaria alternata* [56] and *Talaromyces marneffei* [57].

**Fig. 9:**
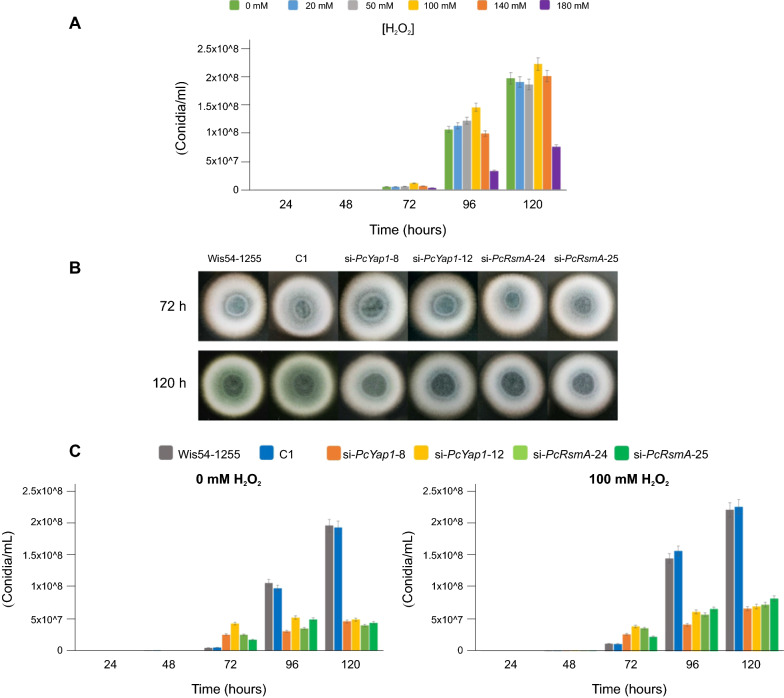
Effect of H_2_O_2_-induced oxidative stress and of PcYap1 and PcRsmA attenuation on conidia production. **A** Conidia production in strain *P. chrysogenum* Wis54-1255 grown on Power medium with different concentrations of H_2_O_2_. The H_2_O_2_ was added at the moment of pouring the media onto the plates, next, 50 µL of a suspension with 1 × 10^6^ conidia/mL was spread onto the plates, which were incubated for 5 days at 28 °C. **B** Colonies of control strains, *Pc-yap1* and *Pc-rsmA* knockdown strains grown on Power medium for 72 and 120 h at 28 °C. Four microliters of a solution with 1 × 10^6^ conidia/mL were inoculated at the center of Petri dishes. **C** Conidia production in strains with knocked down expression of *Pc-yap1* or *Pc-rsmA*. Strain C1 contains the empty pGpdPki-RNAi vector used for gene silencing. To test the effect of H_2_O_2_-generated oxidative stress 100 mM H_2_O_2_ was added at the moment of pouring the media onto the plates (right panel). In **A** and **C** bar sizes represent the result of three biological replicas, error bars correspond to standard deviation. See “Materials and methods” section for details

We then analyzed how the onset of the conidiation process responded to the presence of different concentrations of H_2_O_2_ in the culture medium (Fig. 9A). Conidiation levels were not significantly affected by the addition of 20, 50, or 140 mM H_2_O_2_, whereas with 100 mM there was a 133%, 37% and 15% increase in the number of produced conidia at 72, 96, and 120 h, respectively.

Next, we analyzed the participation of PcYap1 and PcRsmA in the conidiation process. In medium without H_2_O_2_, knockdown of both *Pc-yap1* and *Pc-rsmA* had a kind of fluctuating effect on conidiation (Fig. 9C). In the control strains, conidiation started at 48 h, while in *Pc-yap1*- and *Pc-rsmA*-knocked down strains it started at 72 h. At this time, the conidia counting in these strains was much higher (3.5- to 9-fold) than in the control strains. However, this initial conidiation-stimulating effect subsides as the conidiation proceeds; in *Pc-yap1*-knocked down strains conidiation nearly stalls at 72 h, whereas in *Pc-rsmA*-knocked down strains the number of conidia increased up to 96 h (approximately doubling in the interval 72–96 h) and then conidiation stopped. In the control strains, conidiation proceeds normally, increasing the conidia counting by more than one order of magnitude between 72 and 96 h and then doubling between 96 and 120 h. The final number of conidia at the end of the cultures in the *Pc-yap1*- and *Pc-rsmA*-knocked down strains was around 23% of the number in the control strains. The addition of 100 mM H_2_O_2_ to the culture media produced some changes in the pattern described above. Conidiation started at 48 h in the *Pc-yap1*- and *Pc-rsmA*-knocked down strains, although the number of conidia was one order of magnitude lower than in the control strains. The initial stimulating effect of the attenuation of both proteins also takes place in this condition but to a lower extent (around 2.8-fold more conidia than in the control strains), and then there is a steady moderate increase in conidia counting from 72 to 120 h. The final number of conidia at the end of the H_2_O_2_-added cultures in the *Pc-yap1*- and *Pc-rsmA*-knocked down strains was around 31% of the number in the control strains. In contrast to the important effect that RNAi-silencing of *Pc-yap1* and *Pc-rsmA* had on conidiation, overexpression of both genes did not result in significant changes in the number of conidia produced at any culture time (Additional file 11).

We can conclude that both PcYap1 and PcRsmA are positive regulators of the conidiation process. Their activity is necessary for normal conidiation kinetics and full completion of the process. Normal (wild type) levels of activity of both proteins are sufficient for conidiation to occur with standard kinetics since overexpression had no effect on conidia production. The addition of 100 mM H_2_O_2_ did not result in drastic changes in the effect that expression attenuation of the genes encoding both proteins had on conidiation. Naturally generated ROS seem to be sufficient to trigger the conidiation process, while the addition of H_2_O_2_ stimulates the process mainly at the onset, but its overall effect was moderate (Fig. 9A). Interpretation of the particular conidiation kinetics of the knockdown strains is difficult, there is an initial stimulation followed by early termination of the process. A possible explanation is that other factors are prevalent at the beginning of the conidiation process in response to cues like nutrient starvation or others; absence or low amounts of PcYap1 or PcRsmA would have a positive effect on these factors, thus resulting in a stimulation of conidiation. Eventually, oxidative stress response factors (PcYap1 and PcRsmA) become the main inducers of conidiation, and then low amounts of these proteins would result in early termination or slowdown of the process.

### PcYap1 regulates *brlA* expression and binds to a TTACTAA sequence in its promoter

**Fig. 10:**
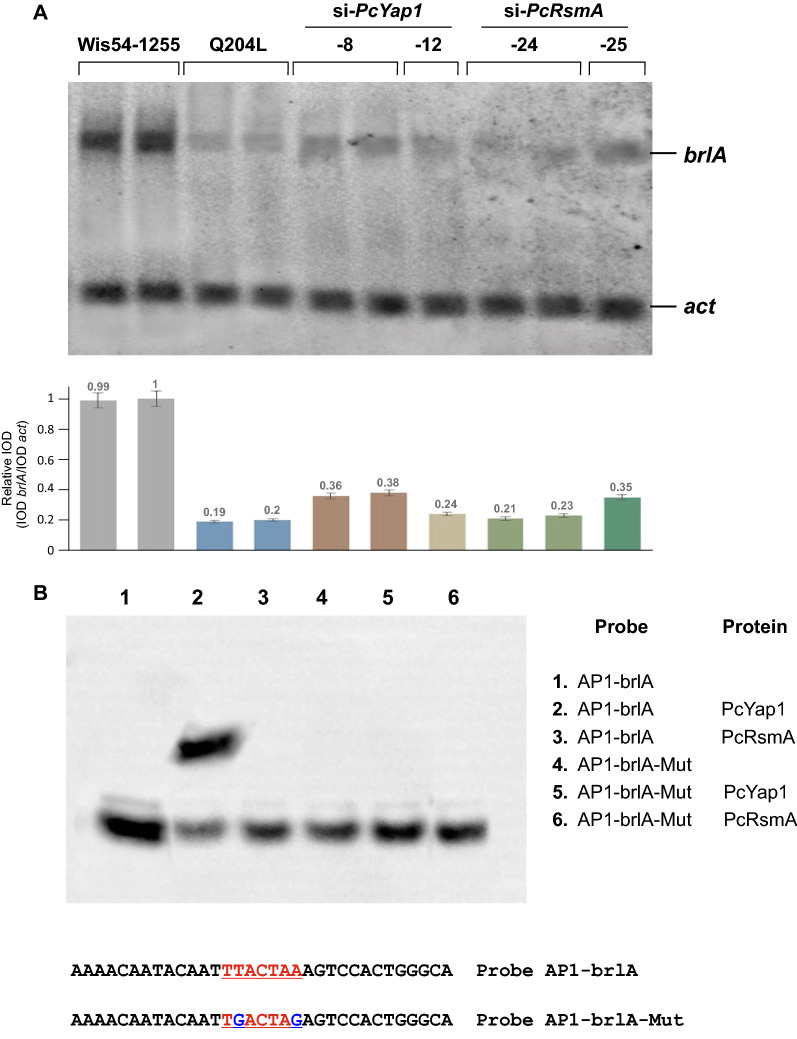
Regulation of the expression of the *brlA* gene by PcYap1 and PcRsmA. **A** Northern blot with RNA extracted from mycelium grown on solid Power medium for 120 h; the probes used were a 573 bp fragment from the *brlA* gene amplified by PCR with primers N-brlA-1F and R, and a 508 bp fragment from the *act* gene amplified by PCR with primers N-actA-1F and R (Additional file 12). The densitometry analysis was performed as described in the legend to Fig. 8. Strain Q204L was used as a control for *brlA* gene transcriptional repression (see text). **B** EMSA of the PcYap1 and PcRsmA binding activities with the probe AP1-brlA, whose sequence corresponds to a region located between − 58 and − 90 bp upstream the ATG start codon of the *brlA* gene promoter, and which contains an AP1 site (highlighted in red); this site is mutated in the AP1-brlA-Mut probe

Once regulation of conidiation by PcYap1 and PcRsmA was confirmed, we decided to investigate if they regulate the transcription of the first gene in the conidiation central regulatory pathway: *brlA*. RNA from control, *Pc-yap1*- and *Pc-rsmA*-knocked down strains was extracted from mycelium grown for 120 h on Power medium, the time of highest expression of *brlA* in *P. chrysogenum* as determined by García-Rico et al. [58]. A strain expressing an activating dominant allele of the gene encoding a Gα subunit of the heterotrimeric G protein (*pga1*^Q204L^) was used as a control since constitutive activation of this pathway results in the nearly absence of conidiation and strong repression of *brlA* expression [58]. Northern blot using a *brlA* probe was performed, and the results showed a clear reduction of *brlA* expression (down to 20–35% of the level of expression in the control strain) in both *Pc-yap1*- and *Pc-rsmA*-knocked down strains (Fig. 10A).

Analysis of the sequence of the *brlA* gene promoter revealed the presence of a typical AP-1 binding site (TTACTAA) 71 bp upstream of the ATG start codon. We performed an EMSA with the purified PcYap1 and PcRsmA proteins using a probe containing this site and another probe with a mutated site: TGACTAG. PcYap1 produced a clear mobility shift with the TTACTAA-containing probe but failed to bind the mutated probe, whereas PcRsmA did not show binding to any of the probes (Fig. 10B).

These results demonstrate that regulation of conidiation by PcYap1 is exerted by regulating the *brlA* gene expression, most likely through binding to the TTACTAA site in its promoter, not excluding the possibility that PcYap1 has additional targets for the regulation of this complex process. In the case of PcRsmA, the regulation of conidiation is very likely mediated by PcYap1 via regulation of its expression (Fig. 8).

## Discussion

PcYap1 is, with a high degree of certainty, the as-then-unidentified transcription factor PTA1 reported by Kosalková et al. [31] to bind to the TTAGTAA regulatory sequence in the *pcbAB* gene promoter. Kosalková et al. [33] proposed that PTA1 may be a complex formed by more than one protein. In this work, we demonstrate that heterologously expressed and purified PcYap1 is able to specifically bind the regulatory sequence TTAGTAA, and that knockdown of *Pc-yap1* causes a decrease in penicillin production. PcYap1 is a typical bZIP transcription factor, orthologous to yeast Yap1 and sharing characteristics with the AP-1 family of transcription factors, whose DNA binding sequences are very similar, and identical in some cases, to the PcYap1-binding site in the *pcbAB* gene promoter. Therefore, we identify PcYap1 with PTA1, and propose that the name PcYap1 be used henceforth since it is more defining and identifying, not excluding the possibility that PcYap1 may interact in vivo with other proteins forming a complex that binds the TTAGTAA sequence, and in such case PTA1 may be used to refer to this, hypothetical yet, complex.

Oxidative stress has gained considerable attention as a triggering agent of different processes in filamentous fungi since it was first proposed that cell differentiation is a response to oxidative stress [59]. More recently, oxidative stress has been linked to the onset of secondary metabolism [12, 60], including β-lactam biosynthesis [61, 62], and some transcription factors mediating the cell response to the presence of ROS have been implicated in the regulation of the biosynthesis of different secondary metabolites [8, 9]. In this work, we have shown that the transcription factors PcYap1 and PcRsmA participate in the oxidative stress-mediated regulation of penicillin biosynthesis, and characterized the function of both proteins in the oxidative stress response of *P. chrysogenum* and in the conidiation process.

Penicillin production in flask cultures of the *P. chrysogenum* Wis54-1255 strain is stimulated by the addition of 100 mM H_2_O_2_ to the culture medium (Fig. 1). The importance of oxidative stress for the regulation of penicillin biosynthesis is supported by the results obtained by Jami et al. [63], who, in a proteomics study, observed an increase in the representation of several proteins involved in oxidative stress defense in a high yield penicillin producer strain with respect to lower yield producers. When the expression of *Pc-yap1* was knocked down, the stimulating effect of H_2_O_2_ did not take place (Fig. 3A). PcYap1 binds to a TTAGTAA sequence that had previously been shown to be an important regulatory element of the *pcbAB* gene expression [31]. These data indicate that PcYap1 directly regulates penicillin biosynthesis by binding to the *pcbAB* gene promoter at the TTAGTAA sequence in response to oxidative stress.

In the case of *Pc-rsmA*-knocked down strains, penicillin production in H_2_O_2_-added medium was lower than in the control strains too. The final amount of accumulated penicillin was in the range of that in the parental strain Wis54-1255 grown without added H_2_O_2_. Stimulation of penicillin production by H_2_O_2_ in the *Pc-rsmA*-knocked down strains is lower than in the control strains, but not completely abolished as in the case of the *Pc-yap1*-knocked down strains. PcRsmA binds specifically to the sequence TGAGACA, located 68 bp upstream from TTAGTAA in the *pcbAB* gene promoter, and to the sequence TTACGTAA in the *penDE* gene promoter. In vivo functionality of these sequences has not been tested yet. However, in a deletion screen analysis of the *pcbAB* gene promoter performed by Kosalková et al. [31], deletion of the region where the TGAGACA sequence is located resulted in an important decrease in promoter activity. These results strongly suggest that PcRsmA also regulates penicillin biosynthesis in response to oxidative stress by binding to the above-mentioned sequences. The role of RsmA in the regulation of secondary metabolism has been well established for several fungal species, including *A. nidulans* [42, 44], *A. fumigatus* [45], *A. flavus* [64] and *P. fici* [46].

Normal (wild type) levels of both PcYap1 and PcRsmA seem to be sufficient to positively regulate penicillin biosynthesis in response to oxidative stress, since overexpression of either gene had little effect on production, with only a moderate increase at the start of the production time (Fig. 3C and D). Penicillin production started earlier in both the control and the *OE* strains when 100 mM H_2_O_2_ was added to the culture, but overexpression of either protein did not cause an earlier production in the absence of H_2_O_2_. This result can be explained if we consider that induction by ROS is probably necessary for PcYap1 and PcRsmA to become active and exert their function on transcription activation of the penicillin genes. Overexpression will not necessarily produce a specular reflection of the effects caused by silencing; i.e. higher amounts of the proteins in the cell would have no effect if they are not activated.

bZIP-type transcription factors may regulate SM production in two ways, by direct binding to promoters of structural or regulatory genes in a cluster, as shown for AtfB [13, 23] and RsmA [44], or by modifying intracellular ROS levels through activation of the antioxidant defense system, as demonstrated for Yap1 orthologs in *A. parasiticus* [21], *A. ochraceus* [40], *A. nidulans* [47] and *F. graminearum* [41]. Our results confirm the role of PcRsmA as a transcription factor binding to promoters of SM genes to regulate SM production and establish for the first time a direct role of a Yap1 ortholog in the transcriptional regulation of an SM gene by binding to a specific regulatory sequence in its promoter. Thus, the role of Yap1 in the biosynthesis of the toxins aflatoxin and trichothecene is opposite to that in penicillin biosynthesis. In the latter case, Yap1 directly stimulates transcription of the *pcbAB* gene by binding to the regulatory sequence TTAGTAA, resulting in increased levels of penicillin, whereas in the case of aflatoxin and trichothecene the effect is indirect by means of controlling the amount of ROS by induction of the antioxidant defense system, which results in downregulation of toxin production, as revealed by the higher toxin accumulation observed in Δ*yap1* mutants [21, 40, 41] and decreased production in *OE::yap1* strains [41, 47]. Interestingly, in a transcriptomics analysis of conidia from an *A. nidulans* strain with a deletion of the *napA* gene (*Yap1* ortholog), Mendoza-Martínez et al. [65] found downregulation of the penicillin biosynthetic gene *ipnA* (= *pcbC*) (-3.25-fold) and some other secondary metabolism-related genes with respect to the wild type, which suggests a positive regulation of penicillin biosynthesis and other secondary metabolites by NapA. The authors proposed that in *Aspergillus*, secondary metabolism may be regulated by NapA in opposite ways during growth and conidiation. Penicillin production has not been tested in *A. nidulans napA* mutants, and there are no canonical Yap1 binding motifs in the *ipnA* gene promoter. Therefore, it is still unclear whether the effect of the *napA* gene deletion on the expression of the *ipnA* gene is direct or indirect through some other regulatory mechanism.

Veiga et al. [66] reported that the transcript levels of *Pc-rsmA* were seven-fold higher in *P. chrysogenum* Wis54-1255 than in the penicillin high-producing strain DS17690 using a microarray approach, which is in apparent conflict with our findings about PcRsmA being a positive regulator of penicillin biosynthesis. Industrial strains are selected for high production in bioreactors, where conditions are different from cultures in flask. In these conditions, the role of PcRsmA may not be very relevant for penicillin biosynthesis, for instance if the bioreactor process-generated oxidative stress is dealt with by industrial strains in a way that makes the PcRsmA role different or irrelevant. In fact, changes in the oxidative stress response have been observed in a high-producing strain with respect to Wis54-1255 in a comparative proteomic analysis [63]. Other possibilities are that the *Pc-rsmA* gene had been mutated during the strain improvement program rendering it inactive or altered in its functionality in strain DS17690, or that, also as a result of strain improvement, other regulatory networks may have bypassed the need for PcRsmA to induce high expression levels of the penicillin genes in this strain.

The bZIP transcription factor AtfB/Atf21 was another candidate for oxidative stress-mediated regulation of the penicillin genes since it has been reported to mediate both oxidative stress response and secondary metabolism in some *Aspergillus* species [13, 23, 48, 49]. AtfB belongs to the ATF/CREB family of transcription factors, which bind as homo or heterodimers to the consensus sequence TKACGTMA [50]. AtfB was first identified in *A. oryzae* as a transcription factor with bZIP and basic domains characteristic of the cyclic AMP-response element-binding protein (CREB) family [49]. Later, an AtfB ortholog was cloned and characterized in *A. parasiticus* by Roze et al. [23] based on the *A. flavus* AFLA_094010 gene, whose deduced amino acid sequence (XP_002381221) shared 96% identity to both *A. oryzae* and the newly sequenced *A. parasiticus* AtfB. The AtfB proteins in these three species did not show high identity with other fungal proteins; they show 9–10% identity to Atf21 of the fission yeast, 53% to an *A. fumigatus* putative transcription factor Atf21, and less than 45% to similar proteins from other fungi. Hence Roze et al. [23] concluded that AtfB may be unique to these three *Aspergillus* species. The highest match to AtfB in the genome of *P. chrysogenum* was the protein encoded at locus Pc21g08330, which shows 45.8% identity head-to-tail to AtfB from these species. We have named this protein PcAtf21. In *A. parasiticus*, AtfB binds to a region in the promoter of the aflatoxin gene *nor-1*; this binding requires both an AP-1-like (TGAGTAC) and a CRE-like site, named CRE1 (TGACATAA), which are adjacent and separated by 12 bp [23]. In addition, Roze et al. [23] found a consensus sequence (AGCCS) located immediately upstream of the CRE1 site in five aflatoxin promoters that demonstrated AtfB binding. Neither AP-1-like sites nor AGCCS-like sequences are present in the *penDE* gene promoter close to the CRE site (TTACGTAA), which could explain the inability of *P. chrysogenum* PcAtf21 to bind in vitro to this DNA region while it does bind the probe NorR4 containing the aforementioned motifs (Fig. 5B). More studies will be required to elucidate the binding properties of PcAtf21 and its possible role in oxidative stress response and secondary metabolism regulation.

While PcAtf21 failed to bind a probe containing the TTACGTAA CRE motif, PcRsmA was able to bind the probe, and this binding was suppressed by mutations at positions 1 and 6 (CTACGAAA) of the motif, which indicates that PcRsmA binds specifically to the CRE motif. Therefore, PcRsmA binds two different sequences in the promoters of the penicillin genes: TGAGACA and TTACGTAA. It also binds the sequence TGACACA from a probe belonging to the *A. nidulans aflR* gene promoter (Fig. 4B). Versatility in the binding capacity of RsmA proteins might be a common feature, considering that *A. nidulans* RsmA also possesses the capacity to bind two different sequences in the *aflR* gene promoter: TGACACA and TTAGTAA, in addition to the sequence TTACTAA of another probe from yeast [44].

The CCAAT-binding protein complex AnCF (CBC) coordinates the response to oxidative stress in *A. nidulans* [67]. AnCF binds to the promoter and regulates the expression of *napA* [67] and the penicillin genes by binding to CCAAT regulatory elements [68]. Multiple CCAAT boxes are present in the *pcbAB-pcbC* intergenic region and the *penDE* gene promoter in *P. chrysogenum*. Therefore, it will be of great interest to test if these CCAAT boxes play a role in regulating the expression of the penicillin genes and if this regulation is connected to oxidative stress. In addition, the redox state of the cell and the thioredoxin system have been proposed to play another role in penicillin biosynthesis in *P. chrysogenum*, forming and reducing disulfide bonds that lead to the formation of oxidized disulfide bis-ACV (which cannot be used by isopenicillin N synthase) and free ACV (substrate for this enzyme), respectively [69]. Therefore, penicillin biosynthesis is probably affected by oxidative stress in several different ways.

The role of Yap1 orthologs as mediators in the defense against ROS has been firmly established both in yeasts [15, 70] and filamentous fungi (reviewed by [9, 39]). Yap1 mutant strains show altered ROS levels and expression of genes involved in the defense against oxidative stress. Our results with PcYap1 add to this role of Yap1 proteins (Fig. 6). In the case of RsmA orthologs, their role in general and oxidative stress defense is less consistent. PcRsmA shows the highest identity in the genomes of yeasts to *S. cerevisiae* Yap3 and *C. albicans* FCR3. Little is known about Yap3 function in the cell, it shows virtually no response in genomic microarray analyses to multiple forms of environmental insults and cellular stresses [71], and Yap3-dependent transcription responds to aminotriazole but not to H_2_O_2_ or cadmium [36]. North et al. [55] reported that Yap3 plays a specific role in the cellular response to hydroquinone (HQ). For its part, overexpression of FCR3 confers resistance to fluconazole and 4-nitroquinoline 1-oxide [43]. In filamentous fungi, RsmA proteins do not show a consistent pattern with respect to stress response. In *A. nidulans*, an *OE::rsmA* strain revealed no altered response to antifungals, oxidative stressors or heavy metals as compared to the wild type [44, 47], while in a Δ*rsmA* mutant ROS production was similar to that in the control strain [72]. In contrast, in *A. fumigatus*, strains containing an *OE::rsmA* allele grew better than their respective controls on menadione-containing medium, thus indicating a role of RsmA in the defense against menadione-induced ROS [45]. In *A. flavus*, *AflrsmA*-overexpressing strains showed increased sensitivity to menadione sodium bisulfite (MSB), whereas *AflrsmA* deletion caused less sensitivity to tert-butyl hydroperoxide (tBOOH) [64]. For its part, in *P. fici*, deletion of the *rsmA* ortholog (*PfzipA*) resulted in differential responses to oxidative stress agents: it caused resistance to tBOOH, diamide, and menadione sodium bisulfite, but increased sensitivity to H_2_O_2_ [46]. In this work we demonstrate that PcRsmA is clearly involved in the defense against H_2_O_2_-induced oxidative stress, as shown by the results on ROS accumulation in *Pc-rsmA*-knocked down/overexpressing strains and on conidia viability after exposure to H_2_O_2_ (Fig. 6). PcRsmA is able to sense H_2_O_2_-generated ROS in vitro changing its conformation as revealed by its mobility in gels (Fig. 7). In *A. nidulans*, *rsmA* expression was found to be induced by H_2_O_2_ stress [73], and in a microarray study performed by Emri et al. [74], aiming to analyze genome-wide expression changes caused by various stresses, both *rsmA* and *napA* genes were found to be part of the COSR (Core Oxidative Stress Response) genes. The authors proposed that RsmA and NapA can be a link between the regulation of stress response and secondary metabolite production in *A. nidulans*. Our results fully support this hypothesis in *P. chrysogenum*; both proteins play similar roles in defense against H_2_O_2_-induced oxidative stress and directly regulate the expression of penicillin genes by binding to specific regulatory sequences in their promoters. Both proteins are ROS sensors linking oxidative stress to secondary metabolite biosynthesis.

Oxidative stress is an important triggering agent for differentiation in filamentous fungi [4]. Effects on conidiation have been observed in mutant strains lacking Yap1: conidiation was decreased to half in an *A. nidulans ΔnapA* mutant with respect to the wild type [65], in *A. ochraceus* a reduced number of morphologically larger conidia were formed in a Δ*Aoyap1* strain as compared to the wild type [40], and in *T. marneffei*, a Δ*yapA* mutant strain produced a number of conidia three orders of magnitude lower than the wild type [57]. Opposite results were obtained in a Δ*ApYapA* strain of *A. parasiticus*, in which premature conidiation occurs and a higher number of conidia is produced during the first 96 h of growth [21]. Involvement of NapA in *A. nidulans* conidiation is supported by the results obtained by Yin et al. [47], who found that an *OE::napA* strain produced more conidia than the wild type. Our results indicate that PcYap1 plays an important role in *P. chrysogenum* conidiation, since attenuation of PcYap1 expression results in a reduction of conidia production to one-fourth the number in the parental strain (Fig. 9C). A clear association between Yap1 function and development/conidiation was established by Guo et al. [75] in *Magnaporthe oryzae*. Disruption of the *M. oryzae Yap1* ortholog, *MoAP1*, caused a 30- to 40-fold reduction in conidiation, morphological conidia abnormalities, reduced aerial hyphal growth and loss of pathogenicity, along with excess ROS accumulation. Therefore, MoAP1 was proposed as a stage-specific regulator of development and plant infection. As happens with secondary metabolism, the regulation of conidiation by Yap1 orthologs may be exerted directly by controlling the expression of genes triggering and coordinating development, or indirectly by modifying the amount of ROS in the cell. In the cases when *yap1* disruption or knocking-down has a negative effect on conidiation, which is the norm, direct control of conidiation regulatory genes by Yap1 should be expected. In *M. oryzae*, Guo et al. [75] found that disruption of *MoAP1* caused a severe down-regulation of MoCOS1, a determinant of conidiophore formation, and other genes required for conidiation. Little is known, however, about the regulation of genes involved in conidiation by oxidative stress. In a transcriptomic analysis of *A. flavus* submitted to H_2_O_2_-induced oxidative stress, some development-related genes were found to be up-regulated [48], most interestingly *flbC*, which encodes a transcription factor that, in *A. nidulans*, activates transcription of *brlA*, the first of the genes in the conidiation central regulatory pathway [76]. *brlA* and the other two genes in the central regulatory pathway (*abaA* and *wetA*) were also found to be regulated by SakA, a member of the stress-activated MAPK (SAPK) pathway in *T. marneffei* [77]. In a recent work analyzing the phosphoproteome response to H_2_O_2_ in *A. nidulans*, Carrasco-Navarro and Aguirre [78] found that StuA, a protein required for proper expression of *brlA*, was specifically phosphorylated in the H_2_O_2_-condition, which suggests that phosphorylation plays also a role in the oxidative stress-mediated regulation of conidiation; interestingly, NapA was also phosphorylated in the H_2_O_2_-condition. In this work we describe for the first time a direct regulation of a gene in the conidiation central regulatory pathway, the *brlA* gene, by an oxidative stress defense protein; PcYap1 binds to a TTACTAA sequence in the *brlA* gene promoter and upregulates expression of this gene, thus triggering conidiation.

Regarding the role of RsmA proteins in the formation of conidia and other asexual resistance structures, little information is available yet. In *A. flavus*, *AflrsmA* deletion decreased sclerotia formation [64]. Results in *A. nidulans* are inconclusive; only an *OE::rsmA* strain has been tested for conidia production, showing no conidiation. However, the same profile was observed in the wild type strain in the conditions used [47]. In *A. fumigatus*, no significant differences were observed in spore production between the Δ*rsmA* and *OE::rsmA* strains and their respective controls [45]. In contrast, in *P. chrysogenum*, we have found clear evidence that PcRsmA is a positive regulator of the conidiation process since *Pc-rsmA*-knocked down strains sporulated less than the control strains and within a similar range to the *Pc-yap1*-knocked down strains (Fig. 9C).

**Fig. 11:**
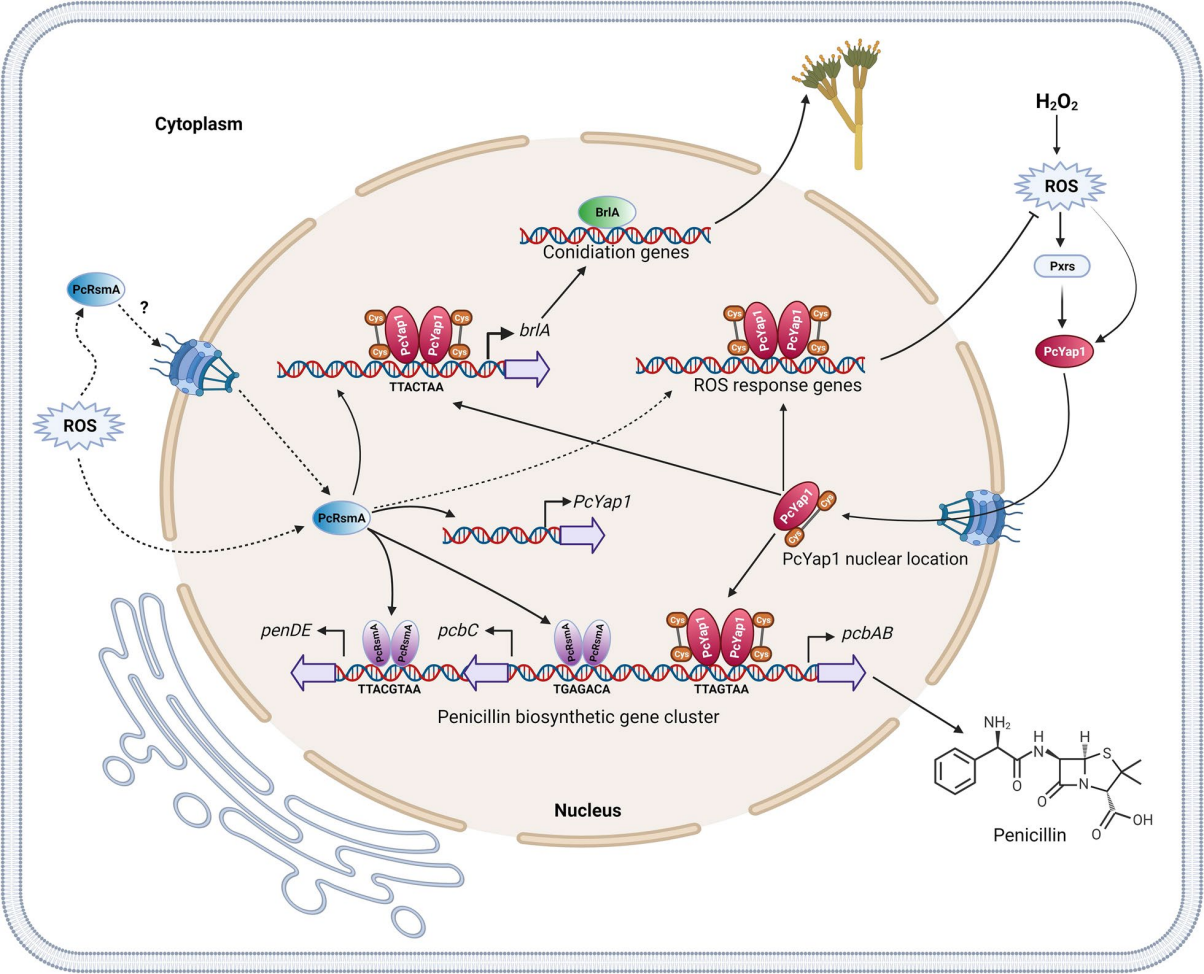
Proposed model for the regulation of penicillin biosynthesis and conidiation in response to oxidative stress by PcYap1 and PcRsmA. The model has been elaborated after the results obtained in this work complemented with previously published results with sufficient evidence to be applicable to this outline. In presence of naturally occurring or H_2_O_2_-generated ROS, PcYap1 undergoes conformational change, by direct sensing or mediated by peroxiredoxins (Prxs), and locates to the nucleus (see text). In these conditions, PcRsmA senses ROS, probably undergoes conformational change and locates to the nucleus by an unknown mechanism. PcYap1 binds a previously described regulatory sequence in the *pcbAB* gene promoter (TTAGTAA), while PcRsmA binds to a site 68 bp upstream from the PcYap1-binding site and to a second site in the *penDE* gene promoter. The two transcription factors positively regulate penicillin biosynthesis. PcRsmA positively regulates transcription of the *Pc-yap1* gene. PcYap1 binds to an AP-1 site in the conidiation central regulatory pathway gene *brlA* and induces its transcription. PcRsmA also stimulates transcription of *brlA*, through direct (unproven) binding to the promoter or via regulation of *Pc-yap1* transcription, or both. Upregulation of the *brlA* gene triggers conidiation. Both PcYap1 and PcRsmA participate in oxidative stress defense, reducing the amount of ROS in the cell; PcYap1 by directly controlling transcription of several oxidative stress response genes, and PcRsmA by putatively controlling expression of oxidative stress response genes or through regulation of *Pc-yap1* transcription, or both. See text for additional details. Created with BioRender.com

It is noteworthy that the concentration of H_2_O_2_ that causes a higher stimulation of penicillin production, 100 mM, is the same that induces an increase in conidia production (Figs. 1 and 9). This fact points to a coordinated regulation of secondary metabolism and differentiation by the presence of ROS within a certain concentration range. The results obtained in this work indicate that both PcYap1 and PcRsmA play an important role in this coordination, linking oxidative stress to secondary metabolite production and conidiation. Besides, the functions of both proteins in the cell seem to be largely overlapping, at least concerning the processes studied in this work, and both proteins respond similarly to the presence of the same H_2_O_2_ concentrations in vitro (Fig. 7). PcYap1 and PcRsmA might have similar targets in the genome; this actually happens in the penicillin gene cluster, where there is one binding site for PcYap1 and two sites for PcRsmA. The fact that PcRsmA regulates the expression of *Pc-yap1* must also contribute to this trait. We have elaborated a model for the cellular processes regulated by PcYap1 and PcRsmA that have been described in this work (Fig. 11).

Oxidative stress is an important factor in industrial processes in bioreactors [79], hence the interest in characterizing the oxidative stress response and its influence on SM production in industrially important fungi, which may help us to develop more stress-tolerant strains and improve production [80]. The findings obtained in this work may also have applications in the development of *P. chrysogenum* strains as heterologous expression systems [81], by means of using cis-acting regulatory elements to increase transcription of engineered promoters modulated by oxidative stress. Our results increase the knowledge on the regulation of the penicillin biosynthetic genes, a model for secondary metabolism gene regulation studies.

## Conclusions

bZIP transcription factors PcYap1 and PcRsmA respond to the presence of H_2_O_2_-generated ROS and regulate oxidative stress response in the cell. Both proteins mediate ROS regulation of penicillin biosynthesis and conidiation by binding to specific regulatory elements in the promoters of key genes.

PcYap1 binds to a previously identified regulatory sequence in the promoter of the penicillin gene *pcbAB* (TTAGTAA), thus revealing the identity of the binding protein named PTA1 by Kosalková et al. [31]. This is the first report of a Yap1 protein directly regulating transcription of a secondary metabolite gene.

PcYap1 binds to a TTACTAA sequence in the promoter of the first gene of the conidiation central regulatory pathway: *brlA*, thus regulating its expression and the conidiation process.

PcRsmA regulates penicillin biosynthesis and binds to the sequences TGAGACA and TTACGTAA (CRE motif) in the promoters of the genes *pcbAB* and *penDE*, respectively. PcRsmA also regulates transcription of the *brlA* and *Pc-yap1* genes.

## Materials and methods

### Strains and growth conditions

*P. chrysogenum* Wisconsin 54-1255 (also named Wis54-1255) (ATCC 28089) was used as a recipient for genetic transformation with plasmids derived from the silencing vector pGpdPki-RNAi, and as a control in the characterization of strains with knocked down expression of *Pc-yap1* and *Pc-rsmA*. *P. chrysogenum* Wis54-1255 *pyrG*, auxotroph of uridine, was obtained from strain Wis54-1255 by random mutation with N-methyl-N-nitro-N-nitrosoguanidine [82]; it was used as a recipient for genetic transformation with plasmids derived from the vector pBKSpyrG and as a control in the characterization of strains overexpressing the genes *Pc-yap1* and *Pc-rsmA*. *P. chrysogenum* Q204L was obtained by transformation of strain Wis54-1255 with a plasmid carrying a dominant activating allele of the *pga1* gene (*pga1*^Q204L^), which encodes a Gα subunit of the heterotrimeric G protein of *P. chrysogenum* (Zúñiga-León et al. unpublished results); it was used as a control in Northern blot for transcriptional repression of the *brlA* gene. All *P. chrysogenum* strains were stored at − 20 °C in 40% glycerol, and grown on plates with Power medium [58] for 7 days at 26 °C to collect spores for inoculation of submerged cultures. For the growth of strain Wis54-1255 *pyrG*, the media were supplemented with uridine at a final concentration of 100 µg/mL.

*P. pastoris* X-33 was used for heterologous expression of *P. chrysogenum* proteins. It was stored at − 20 °C in 40% glycerol and maintained in YPD agar medium. *Escherichia coli* DH5α was used as a host for plasmid constructions. *Micrococcus luteus* ATCC-9341 was used in bioassays to quantify penicillin concentration.

### Cultures for penicillin production

Conidia collected from cultures on plates with Power medium were inoculated into 100 mL of complex seed (CS) medium [83] in flasks, at a final concentration of 5 × 10^6^ conidia/mL. The cultures were incubated for 24 h at 250 rpm and 28 °C. Eight millilitres from the seed cultures were then inoculated, in triplicate, into flasks containing 50 mL of complex production (CP) medium (gxL^−1^: lactose, 55; corn steep solids, 35; CaCO_3_, 10; KH_2_PO_4_, 7; MgSO_4_-7H_2_O, 3; potassium phenylacetate, 4; pH 6.1), which were incubated for 120 h at 250 rpm and 28 °C. Every 12 or 24 h, 2 mL samples were taken to determine penicillin G and dry weight as previously described [84, 85]. For cultures submitted to oxidative stress, H_2_O_2_ was added to the CP medium at the time of inoculation at a final concentration of 25, 50, 100, 150 and 200 mM.

### Quantification of reactive oxygen species (ROS)

Cultures were performed as indicated above for penicillin production. For the determination of ROS in mycelium from control and 100 mM H_2_O_2_-added cultures we followed the method described by Miranda et al. [12] with some modifications. Every 24 h, 2 mL samples were taken from the CP medium. Each sample was divided in two 1-mL microtubes, centrifuged for 10 min at 14,000 rpm, and the pelleted biomass was resuspended in 1 mL of cold PBS. To one tube, 10 µM of H_2_DCF-DA (2',7'-dichlorodihydrofluorescein diacetate) was added in the dark, and the tube was maintained on ice for 40 min, the other tube was a background control without H_2_DCF-DA. From each sample, 200 µL were put in a 96-well microplate, in triplicate, and the absorbance reading was adjusted to 485 nm excitation and 530 nm emission in a DTX 880 multimode plate reader (Beckman-Coulter). The obtained signal was corrected by subtracting the background signal and normalized with the biomass (dry weight).

### Analysis of conidiation

Fifty microliters of a suspension with 1 × 10^6^ conidia/mL were inoculated on the center of a Petri dish with Power medium, which was incubated at 28 °C for 120 h. Every 24 h conidia were collected by adding 5 mL of NaCl 0.9% and scraping the surface, then they were centrifuged, concentrated to a final volume of 1 mL, and diluted for counting in a Neubauer chamber. The results were expressed as conidia collected per mL of solution. For cultures submitted to oxidative stress, H_2_O_2_ was added to the Power medium at a final concentration of 20, 50, 100, 140, and 180 mM at the time of casting the medium onto the plates.

### Oxidative stress resistance tests for conidia

One hundred microlitres from a suspension with 1 × 10^4^ conidia/mL was mixed with 900 µL of a solution of NaCl 0.9% containing different concentrations of H_2_O_2_: 0, 100, 130, 160, and 180 mM. The mixture was incubated for 20 min at room temperature and a volume of 50 µL was inoculated on the surface of PDA dishes, which were incubated at 28 °C for 24 h, after which grown colonies were counted.

### Genes and proteins used in BLAST searches, sequence alignments and primer design

The sequences of bZip transcription factors AP-1/Yap1 from *A. fumigatus* (GenBank accession no. XP_750882.1; UniProt: Q4WMH0), Fcr3 (gene ANIA_04562), RsmA from *A. nidulans* (GenBank accession no. XP_662166.1; UniProt: Q5B4G8), and Atf21/AtfB from *A. flavus* (GenBank accession no. XP_002381221.1; UniProt: B8NLU5) were used as query to perform BLAST searches to find their putative homologs in the *P. chrysogenum* genome (*P. chrysogenum* Wis54-1255, aka *P. rubens* Wis54-1255, taxid 500485). (See text).

Other protein sequences used for BLAST searches and sequence alignments were: AP-1-like transcription factor YAP1 (NP_013707; P19880) and AP-1-like transcription factor YAP3 (NP_011854; P38749) from *S. cerevisiae*; transcription factor Pap1/Caf3 (NP_593662; Q01663) and Atf-CREB family transcription factor Atf21 (NP_595707; P78962) from *S. pombe*; fluconazole resistance protein FCR3 (AAL35299; Q8X229) from *C. albicans*; AP-1-like transcription factor NapA (XP_680782; Q5AW17) and bZIP transcription factor Atf21 (CBF78271; Q5AST7) from *A. nidulans*; bZIP transcription factor Fcr3 (AfRsmA) (XP_749389; Q4WIA4) and bZIP transcription factor Atf21 (KMK62786; A0A0J5Q1E3) from *A. fumigatus*; bZIP transcription factor Atf21 (XP_001274576; A1CBN2) from *A. clavatus*; basic leucine zipper (bZIP) transcription factor AtfB (XP_001824132; Q2U616) from *A. oryzae*; CRE1 binding protein/basic leucine zipper (bZIP) transcription factor AtfB (ADZ06147; A0A0F0IP79) from *A. parasiticus*; hypothetical protein FGSG_08800 (Fgap1) (XP_011319920; I1RWW4) from *F. graminearum*.

For the designing of primers of the *act* (gamma-actin) and *brlA* genes of *P. chrysogenum* the sequences Pc20g11630 and Pc06g00470 in GenBank were used, respectively.

### DNA extraction

Total DNA from *P. chrysogenum* was obtained from mycelium grown in MPPY medium [85]. The mycelium was powdered with liquid nitrogen in a mortar and DNA extraction was performed as described by Fierro et al. [86]. Alternatively, for extraction of small amounts of DNA, the mycelium was broken in 2-mL tubes with 0.5-mm glassbeads using a MINIBEADBEATER (Biospec) for 3 min, and DNA extraction was performed with the Wizard Genomic DNA Purification kit (Promega) following the manufacturer’s indications.

### Cloning of* Pc-yap1*,* Pc-rsmA* and* Pc-atf21* from the *P. chrysogenum* Wis54-1255 genome

Amplification of the *Pc-yap1*, *Pc-rsmA* and *Pc-atf21* genes containing their respective promoters and terminators was performed by PCR using primers Yap1-F and -R, RsmA-F and -R, AtfB-F and -R (Additional file 12). The amplified fragments were digested with the appropriate restriction enzymes for the restriction sites present at the 5’-ends of the primers and inserted into the previously digested pBluescript KS + vector. (See text for the identity of the genes in the *P. chrysogenum* genome).

### Construction of plasmids for RNAi-mediated silencing and overexpression of the *Pc-yap1 *and *Pc-rsmA* genes

RNAi-mediated gene silencing is an efficient method to knock down the expression of genes in *P. chrysogenum* [83, 87, 88]. We followed a strategy based on the generation of dsRNA as described by Ullán et al. [87] and Cepeda-Garcia et al. [83], using the vector pGpdPki-RNAi (Marcial-Quino, Miranda, Fierro, unpublished) (Additional file 1). Primers siYAP1-F and -R, and siRSMA-F and -R, were used for PCR amplification of fragments from the *Pc-yap1* and *Pc-rsmA* genes, respectively (see Additional file 1 for details). These fragments were inserted into the silencing vector pGpdPki-RNAi at the NcoI site located between the two opposite constitutive promoters (*A. nidulans gpd* and *Aspergillus niger pki*), to obtain the silencing plasmids pGpdPki-RNAi/PcYap1 and pGpdPki-RNAi/PcRsmA.

For gene overexpression, the *pki* constitutive promoter was fused to a DNA fragment containing the ORF plus 300 bp downstream the stop codon (thus comprising the terminator) from each of the two genes. A strategy based on recombinant PCR was used for the fusion at the ATG start codon (Additional file 2). For the construction of plasmid pPyrG-*pki::Pc-yap1*, the *pki* promoter was amplified with primers pki-PcYap1-F and -R (using plasmid pGpdPki-RNAi as template), obtaining a fragment of 1041 bp. The *Pc-yap1* gene was amplified with primers PcYap1-Ter-F and -R to obtain a fragment of 2181 bp. The product of both PCRs was mixed and submitted to one PCR cycle with the conditions: 5 min at 95 °C, 5 min at 57 °C and 10 min at 72 °C, and then to 30 cycles with the conditions: 30 s at 95 °C, 1 min at 57 °C, 4 min 72 °C. *pfu* Ultra II Fusion HS DNA Polymerase (Agilent) was used in all reactions. For the construction of plasmid pPyrG-*pki::Pc-rsmA* the same steps were followed, using primers pki-RsmA-F and -R, and RsmA‐Ter‐F and ‐R (Additional file 12). The final amplified fusion fragments were digested with the appropriate restriction enzymes (see legend to Additional file 2) and inserted into the vector pBKSpyrG (pBluescript-derived containing the *pyrG* gene of *P. chrysogenum*).

### Transformation of *P. chrysogenum*

*P. chrysogenum* Wis54-1255 was the recipient for transformation with plasmids pGpdPki-RNAi/PcYap1 and pGpdPki-RNAi/PcRsmA for gene silencing. The transformation was performed as described by Cantoral et al. [89] using phleomycin at a concentration of 30 µg/mL as selection marker when regenerating the protoplasts on Czapek minimal medium supplemented with 1 M sorbitol. Colonies were then submitted to transfers in different media under selective pressure to ensure the stability of transformants (Power to get conidia – Czapek after dilution to single conidia and plating – Power to grow the final homokaryons).

*P. chrysogenum* Wis54-1255 *pyrG* was the recipient for transformation with plasmids pPyrG-*pki::Pc-yap1* and pPyrG-*pki::Pc-rsmA* for gene overexpression. The transformation was performed as described by Díez et al. [82] using auxotrophy of uridine as selection marker when regenerating the protoplasts on Czapek minimal medium supplemented with 0.7 M KCl. Colonies were then submitted to the same transfers described above.

### Confirmation and selection of *P. chrysogenum* transformants with knocked down expression and overexpression of genes *Pc-yap1* and *Pc-rsmA*

Out of a total of 50 transformants with plasmid pGpdPki-RNAi/PcYap1, we chose eight based on similar macroscopic phenotypes between them, which were named Y1, Y3, Y4, Y5, Y8, Y11, Y12, Y18. The same procedure was followed for the transformants obtained with plasmid pGpdPki-RNAi/PcRsmA, which were named R24, R25, R30, R31, R32, R33, R34, R39. The presence of the silencing plasmids in these transformants was confirmed by PCR (Additional file 3). Preliminary assays with these transformants were carried out consisting of analysis of phenotypic features (rate of conidiation, resistance to ROS) and non-quantitative RT-PCR. From the obtained results, we chose two transformants from each gene: Y8, Y12, R24 and R25 (named as strains si-*PcYap1*-8, si-*PcYap1*-12, si-*PcRsmA*-24 and si-*PcRsmA*-25), which showed typical, average and stable phenotypes. These strains were submitted to semiquantitative RT-PCR analysis to confirm knocked-down expression of the *Pc-yap1* and *Pc-rsmA* genes (Additional file 3E). The results showed undetectable amounts of *Pc-rsmA* transcript in both si-*PcRsmA*-24 and si-*PcRsmA*-25 strains, and around 28% *Pc-yap1* transcript with respect to the amount in the parental Wis54-1255 strain in both si-*PcYap1*-8 and si-*PcYap1*-12 strains. These strains were thus selected to study the function of the PcYap1 and PcRsmA proteins in the cell.

A similar approach was followed for transformants obtained with the overexpression plasmids pPyrG-*pki::Pc-yap1* and pPyrG-*pki::Pc-rsmA*. The presence of the plasmids in the transformants was confirmed by PCR (Additional file 4). Two transformants with each overexpressed gene, showing average and stable phenotypes, were chosen for further experiments; they were named: *OE::PcYap1-A*, *OE::PcYap1–E*, *OE::PcRsmA-C* and *OE::PcRsmA-H*.

### RNA extraction

Mycelium grown in submerged cultures was collected by filtration, washed with 0.9% NaCl, dried in filter paper and powdered with liquid nitrogen in a mortar. RNA was extracted with Trizol™ reagent (Ambión, Life Technologies) following the manufacturer’s specifications. The RNA was stored at − 80 °C until use.

For mycelium grown on solid agar media the following procedure was used. Discs of 3MM paper (Whatman) were placed on top of Petri dishes with Power medium, and a solution with 1 × 10^6^ conidia was spread onto its surface. The plates were incubated at 28 °C in an oven for 120 h. The mycelium was collected by scraping the surface of the 3MM paper with a sterile steel spatula and powdered with liquid nitrogen in a mortar. RNA was extracted as indicated above.

### Northern blot

For Northern blot, a protocol was elaborated based on the standard Northern blot technique with radioactive probes [90] but using chemiluminescence instead. After RNA extraction from mycelium grown on Power medium, the RNA was quantified in an Epoch microplate reader (BioTek Instruments). A volume containing 10 µg of RNA was mixed (1:1) with standard RNA gel loading buffer (1.25X) and loaded into a denaturing 1.2% agarose gel in MAE buffer 1X (MAE 10X: MOPS 0.2 M, EDTA 50 mM, pH 7) with 3% formaldehyde; running buffer was MAE 1X. Blotting to a Hybond-N^+^ membrane (GE Healthcare) was made by capillarity with SSC 10X buffer for 12–16 h, after which the RNA was fixed to the membrane in a UVC 500 Crosslinker (Amersham Bioscience) adjusted to 1200 (956 J for 15 s). The probes were labelled with the Biotin DecaLabel DNA Labeling kit (Thermo Scientific) and quantified in an Epoch microplate reader to adjust the concentration to values in the order of ng/µL. Prehybridization of the membrane was performed in a glass container with hybridization buffer (SSC 6X, SDS 0.1%, formamide 40%, Denhart 1X) containing 500 µg/mL salmon sperm DNA (Sigma-Aldrich) for 3 h at 42 °C. This solution was removed and new hybridization buffer containing 100 µg/mL salmon sperm DNA and 15 ng/mL of each labelled probe (previously denatured by boiling for 5 min) was added; the membrane was then incubated for 12 h at 42 °C. The hybridization buffer was removed and the membrane was successively washed with washing buffer I (SSC 2X, SDS 0.1%) at room temperature, washing buffer I at 42 °C, washing buffer II (SSC 0.1X, SDS 0.1%) at 42 °C, and washing buffer II at 65 °C, all washing steps for 15 min. The membrane was left to dry and signal detection was carried out with the Chemiluminescent Nucleic Acid Detection Module (Thermo Scientific) in a ChemiDoc™ MP Imaging System (Bio-Rad) using the Chemiluminescent channel. The relative density of the hybridization signals was determined with the ImageLab 6–0.1 software (Bio-Rad).

### Isolation of the *Pc-yap1, Pc-rsmA* and* Pc-atf21 CDS* and insertion in expression vectors for *P. pastoris*

The CDS of the three genes for heterologous expression of the proteins in *P. pastoris* was obtained following different procedures (Additional file 5). For *Pc-rsmA*, a recombinant PCR approach was followed for removal of the introns (see legend to Additional file 5). In the case of *Pc-yap1*, total RNA was isolated from mycelium grown in submerged culture, then an RT-PCR was performed with primers Yap-1 and Yap-6 (Additional file 12). The *Pc-atf21* gene contains no introns, so the CDS was directly amplified from DNA, using as template the previously cloned gene in pBluescript KS + , with primers AtfB-1 and AtfB-2 (Additional file 12). The CDS of *Pc-yap1* was inserted in the vector pPICZ-A (Invitrogen) by digestion of the amplified fragment and the vector with the enzymes ApaI and KpnI, whereas the CDS of *Pc-rsmA* and *Pc-atf21* was inserted in the vector pPICZ-B with the enzymes EcoRI and XbaI. In all cases, in-frame insertions were obtained which allowed the expression of *c-myc*-6xHis-tagged proteins from the three genes (Additional file 5); all insertions were confirmed by sequencing. The resulting plasmids were named: pPICZ-A/PcYap1, pPICZ-B/PcRsmA, and pPICZ-B/PcAtf21.

### Transformation of* P. pastoris* and purification of recombinant 6xHis-tagged proteins

Plasmids pPICZ-A/PcYap1, pPICZ-B/PcRsmA and pPICZ-B/PcAtf21 were introduced in *P. pastoris* X-33 by electroporation in a GenePulser Xcell (Bio-Rad) following the indications in the manual for the vectors (Invitrogen). The plasmids were linearized with the enzyme SacI prior to transformation. The selection of transformants was performed on YPD agar medium with 50 µg/mL phleomycin. After incubation for 72–96 h at 30 °C colonies of transformants appeared. One colony of each transformant was inoculated in flasks with 50 mL YPD medium containing 2% glycerol and 75 µg/mL phleomycin and incubated at 30 °C, 200 rpm for 16–18 h (reaching an OD_600_ = 2–6). The cultures were centrifuged for 5 min at 5000 rpm; the cells were resuspended in 100 mL of YP medium with 1% methanol and transferred to 1-L flasks for a new incubation at 200 rpm, 30 °C for 72 h. Methanol was added to the cultures at a final concentration of 1% every 24 h to compensate for the evaporation and thus maintain the induction of the *AOX1* promoter to express the recombinant proteins. The cultures were centrifuged at 10,000 rpm, 4 °C for 5 min, and the cells resuspended in 15 mL of breaking buffer (NaH_2_PO_4_ 50 mM, EDTA 1 mM, glycerol 5%, pH 7.4) containing protease inhibitor (Sigma-Aldrich) and glass beads. The cells were disrupted by vortexing (30 s shaking and 1 min on ice for a total of 45 min) and sonication (20 min in an ice bath at 50/60 HZ intensity in a Bransonic Ultrasonic Cleaner). The lysates were centrifuged at 10,000 rpm, 4 °C for 5 min and the supernatants transferred to 50-mL ultrafiltration units (Amicon Ultra 15, 10 KDa pore size, Merck/Millipore), which were centrifuged at 10,000 rpm, 4 °C for 10 min, washed twice with binding buffer (Na_2_HPO_4_ 50 mM, NaCl 0.5 M, pH 8) and centrifuged until the samples were concentrated to a volume of 1.5 mL.

Purification of the recombinant 6xHis-tagged proteins was performed with the Ni–NTA Spin Kit (Qiagen). A volume of 600 µL Ni–NTA agarose was added to the empty columns; they were centrifuged at 14,000 rpm for 5 min. Then 600 µL of binding buffer was added, the columns were left to equilibrate for 10 min and centrifuged again at 14,000 rpm for 5 min, this step was repeated three times. Finally, 600 µL of the ultrafiltrated protein solutions were added to the columns and left overnight at 13 °C. The proteins were eluted with 600 μL binding buffer containing increasing concentrations of imidazole (10, 20, 50, 200, 300, and 500 mM), centrifuging after each step at 10,000 rpm, 4 °C for 5 min. The eluted solutions were stored at -20 °C. Aliquots were analyzed by SDS-PAGE to confirm purification of the proteins to homogeneity, which was attained with 300–500 mM imidazole (Additional file 6). Elution of PcYap1 with different concentrations of imidazole gave repeatedly two bands of similar size (around 66 kDa), even using extracts from different *P. pastoris* transformants. Analysis by mass spectrometry of the two bands separately excised from the gel showed that both corresponded to PcYap1, so we interpreted the result as formation of dimers or, alternatively, isomeric forms produced during expression in *P. pastoris*.

### Electrophoretic mobility shift assay (EMSA)

Probes for EMSA were obtained either by PCR amplification when the length exceeded 80 bp or by annealing (after labelling) of two complementary oligos when the length was below 60 bp (Additional file 12); for annealing, the oligos were mixed, denatured for 3 min at 90 °C, the solution was cooled 1 °C per minute down to the oligos Tm, then maintained at this temperature for 30 min and finally cooled again down to room temperature. A 5% native polyacrylamide gel was made as follows: mixing of H_2_O, 6.25 mL; TBE 5X, 2 mL; acrylamide/BIS 29:1 at 40%, 1.25 mL; sonication for 10 min, and addition of APS 5%, (500 µL) and TEMED, 6 µL. The gel was casted in a Mini-Protean Tetra Cell (Bio-Rad), left to polymerize and pre-run for 60 min at 100 V with cooled TBE 0.5X. The LightShift Chemiluminescent EMSA kit (Thermo Scientific) was used in all steps of the EMSA process. Labelling was performed with Biotin-11-UTP following the protocol of the Biotin 3’ End DNA Labelling kit part. Reactions were prepared by gently mixing the components in this order: MilliQ H_2_O, 6 µL; binding buffer 10X, 2 µL; glycerol 50%, 4 µL; MgCl_2_ 100 mM, 1 µL; labelled probe (20 fmol), 4 µL; purified protein (10 µg), 3 µL. The reactions were incubated at room temperature for 20 min, then mixed with 5 µL loading buffer 5X and loaded onto the gel. Electrophoresis was carried out at 80 V with cooled TBE 0.5X. Next, the gel was blotted onto a nylon Hybond-N^+^ membrane (previously equilibrated with TBE 0.5X for 10 min) in a Mini Trans-Blot Electrophoretic Transfer Cell (Bio-Rad) with cooled TBE 0.5X at constant 380 mA for 60 min. The membrane was then taken out from the assemblage and excess water was removed with filter paper. The DNA was fixed to the membrane in a UVC 500 Crosslinker (Amersham Bioscience) adjusted to 1200 (956 J for 10 min). Signal detection was performed with the Chemiluminescent Nucleic Acid Detection Module kit (Thermo Scientific) following the manufacturer’s indications.

### Oxidation assays of purified proteins

Protein oxidation assays were performed adapting methodologies previously described by Delaunay et al. [53] and Wood et al. [51]. H_2_O_2_ was added at different concentrations (see Fig. 7) to solutions containing 15 or 25 µg of purified PcYap1::*c-myc*-6xHis and PcRsmA::*c-myc*-6xHis in binding buffer (see above); the mixtures were incubated for 0, 5, 15, or 30 min at room temperature. Samples from these reactions were run in 8–10% SDS-PAGE gels in reducing or non-reducing conditions. For reducing conditions, 20 µL of protein loading buffer (Tris–HCl 0.5 M pH 6.8, 1.25 mL; Glycerol, 2.5 mL; SDS 10%, 2 mL; bromophenol blue 0.5%, 0.2 mL; MilliQ water, 3.55 mL) containing 5% β-mercaptoethanol was added to the samples, which were heated at 90 °C for 5 min and loaded onto the gels. For non-reducing conditions, the samples were mixed with 20 µL of loading buffer without β-mercaptoethanol and they were not heated. Electrophoresis were performed in Mini-Protean Tetra Cell (Bio-Rad) at 150 V for approximately 90 min. The gels were stained with Blue Silver Coomasie [91] and analyzed in a Molecular imager Gel Doc EZ System using White tray (Bio-Rad).

### Mass spectrometry of proteins

From an SDS-PAGE fixed and stained with Blue Silver Coomassie, the bands of interest were cut and deposited in 0.25-mL microtubes previously washed with acetonitrile. The digestion with trypsin was performed with the In-Gel Tryptic Digestion Kit (Thermo Scientific). The samples were concentrated and desalted in Pierce® C18 Spin Columns (Thermo Scientific). A volume of 2 μL of sample was mixed with 2 µL of the matrix (α-cyano-4-hydroxycinnamic acid), and 1 µL of this solution was loaded in duplicate into the plate of a MALDI-TOF mass spectrometer (Bruker). Proteins were identified by peptide fingerprinting.

### Bioinformatic analysis

Basic Local Alignment Search Tool (BLAST) at NCBI was used to retrieve proteins orthologous to the ones studied in this work from the NCBI and EBI databases.

Clustal Omega from the EMBL-EBI (https://www.ebi.ac.uk/Tools/msa/clustalo/) was used for multiple protein alignments [92].

For the prediction of NLS in PcYap1 and PcRsmA we used the cNLS Mapper tool (http://nls-mapper.iab.keio.ac.jp/cgi-bin/NLS_Mapper_form.cgi), which predicts importin α-dependent signals [93].

For prediction of NES we used LocNES (http://prodata.swmed.edu/LocNES/LocNES.php), which predicts signals for CRM1-mediated nuclear export [94].

The STRING tool (STRING Consortium 2021, https://string-db.org/) was used to identify putative interactions of PcYap1 and PcRsmA [95].

### Statistical analysis

For statistical analysis of quantifiable data, the NCSS-PASS-GESS program (NCSS, PASS, USA) was used. Means were submitted to a Tukey–Kramer multiple comparison test to determine statistical significance (set at P < 0.05).

## Supplementary Information

**Additional file 1**. Strategy for silencing of expression of* Pc-yap1* and* Pc-rsmA*. (A) A DNA fragment of 458 bp from the exon 2 of* Pc-yap1* was amplified by PCR with primers siYAP1-F and -R, digested with NcoI and inserted at the NcoI site of plasmid pGpdPki-RNAi to obtain plasmid pGpdPki-RNAi/PcYap1. (B) A DNA fragment of 409 bp from the exon 3 of the* Pc-rsmA* gene was amplified by PCR with primers siRsmA-F and -R, digested with NcoI and inserted at the NcoI site of plasmid pGpdPki-RNAi to obtain plasmid pGpdPki-RNAi/PcRsmA. (C) Strategy of silencing. Transcription from the opposite-oriented promoters* gpd* and *pki* generates complementary RNA strands that form a dsRNA with the sequence of the inserted 458 bp fragment from *Pc-yap1*. This dsRNA will cause silencing of the expression of *Pc-yap1* through the RNAi pathway.

**Additional file 2**. Recombinant PCR for overexpression of genes *Pc-yap1* and* Pc-rsmA*. The *pki* gene promoter from* A. niger* was fused to fragments from the genes extending from the ATG start codon to around 300 bp downstream the TGA stop codon to ensure the presence of the transcriptional terminator. The position of the primers used for PCR reactions is indicated (see Materials and Methods for details). The final fragments with the genes fused to the *pki* promoter were digested with the restriction enzymes EcoRV and SpeI (P*pki*::*Pc-yap1*) or KpnI and XhoI (P*pki::Pc-rsmA*) and inserted in the vector pBKSpyrG to obtain the plasmids p*PyrG-pki::Pc-yap1* and p*PyrG-pki*::*Pc-rsmA*, respectively; the restriction sites in the primers are highlighted in color. Introns are indicated in grey color. Added restriction sites at the 5’-end of the primers are highlighted in orange color.

**Additional file 3**. Confirmation of the presence of plasmids for RNAi-mediated silencing of *Pc-yap1* and* Pc-rsmA* and analysis of silencing in* P. chrysogenum* transformants. (A) Close-up of the pGpdPki-RNAi vector region with the opposite-oriented* gpd* and *pki* promoters, and the plasmids pGpdPki-RNAi/PcYap1 and pGpdPki-RNAi/PcRsmA with the inserted DNA fragments from the *Pc-yap1* and* Pc-rsmA* genes at the NcoI site. Primers Gpd1(pki1)R and Pki1(gpd1)F are shown at the position of annealing with sequences in the* gpd* and *pki* promoters, respectively. The expected size of amplified DNA fragments in each type of transformant is indicated with double-headed arrows. (B) Agarose gel with the result of PCR amplification with primers Gpd1(pki1)R and Pki1(gpd1)F using as template DNA from the purified pGpdPki-RNAi vector (lane V), DNA from strain C1 containing the pGpdPki-RNAi vector (lane C1) and DNA from strain Wis54-1255 (lane W). (C) Results of the PCR amplification performed with the mentioned primers and DNA from eight transformants with the pGpdPki-RNAi/PcYap1 plasmid (Y1…. Y18). (D) Results of the PCR amplification performed with the mentioned primers and DNA from eight transformants with the pGpdPki-RNAi/PcRsmA plasmid (R24… R39). (E) Silencing of expression of *Pc-yap1* (upper panels) and* Pc-rsmA* (lower panels) in selected strains containing the RNAi-silencing plasmids pGpdPki-RNAi/PcYap1 and pGpdPki-RNAi/PcRsmA, respectively. RNA was extracted from mycelium grown for 60 h in MPPY medium and used for semiquantitative RT-PCR (as described by Domínguez-Santos et al. [96]) using primers qPcYap1-F and -R for analysis of *Pc-yap1* expression, qRsmA-F and -R for* Pc-rsmA*, and qactA-F and -R for* act*. The left panels show the intensity of the bands in an agarose gel loaded with the products of the RT-PCR reactions, and the right panels the densitometry analysis of the bands. The results were normalized with the bands of the constitutively expressed* act* gene. The parental strain Wis54-1255 (lane W) was used as reference with a value for integrated optical density (IOD) of 100. Y8: strain si-*PcYap1*-8; Y12: si-*PcYap1*-12; R24: si-*PcRsmA*-24; R25: si-*PcRsmA*-25.

**Additional file 4**. Confirmation of the presence of plasmids for overexpression of *Pc-yap1* and* Pc-rsmA* in *P. chrysogenum* transformants. (A) Scheme of the fusion of the *pki* promoter with the *Pc-yap1* (top) and* Pc-rsmA* (down) genes at the ATG start codon in the plasmids pPyrG-pki::*Pc-yap1* and pPyrG-pki::*Pc-rsmA*. Primers Pki1(gpd1)F, OE-PcYap1-R, siYAP1-R, OE-RsmA-R and siRSMA-R are shown at the position of annealing with sequences in the *pki* promoter and the *Pc-yap1* and* Pc-rsmA* genes, respectively. The expected size of amplified DNA fragments in each type of transformant for every primer pair is indicated with double-headed arrows. (B) Agarose gel with the result of PCR amplification with primers Pki1(gpd1)F and OE-PcYap1-R using as template DNA from the purified pPyrG-*pki*::*Pc-yap1* plasmid (lane P), DNA from strain Wis54-1255 (lane W), a transformant with the empty pBKSpyrG vector (lane Y) and transformants* OE::PcYap1-A* through* –F* (lanes A through F). Transformants selected for further characterization are indicated with a red circle. (C) Result of PCR amplification with primers Pki1(gpd1)F and OE-RsmA-R using as template DNA from transformants* OE::PcRsmA-A* through –*H* (lanes A through H). (D) Result of PCR amplification with primers Pki1(gpd1)F and siRSMA-R using as template DNA from the purified pPyrG-*pki*::*Pc-rsmA* plasmid (lane P), total DNA from strain Wis54-1255 (lane W), a transformant with the empty pBKSpyrG vector (lane Y) and transformants *OE*::*Pc-rsmA*-*C* through –*H* (lanes C through H). Transformants selected for further characterization are indicated with a red circle.

**Additional file 5**. Isolation of *Pc-yap1*,* Pc-rsmA* and* Pc-atf21* CDS and insertion in expression vectors for *P. pastoris.* (A) An RT-PCR was performed using RNA from a submerged culture and primers Yap-1 and Yap-6, which contain restriction sites for KpnI and ApaI, respectively. The amplified fragment was inserted in the pPICZ-A vector after digestion with these enzymes and ligation. The in-frame insertion is shown, indicating the position of the cloning enzymes, the *Pc-yap1* CDS and the* c-myc*-6xHis tag. (B) For* Pc-rsmA*, a recombinant PCR approach was followed, designing primers with sequences linking exon 1 to 2 (RsmA-2 and RsmA-3) and exon 2 to 3 (RsmA-4 and RsmA-5), plus primer RsmA-1, annealing at the start of the ORF and containing an EcoRI restriction site at 5’, and primer RsmA-6, annealing at the end of the ORF and containing an XbaI restriction site. PCR reactions were performed to separately amplify exon 1 (primers RsmA-1 and -2), exon 2 (primers RsmA-3 and -4) and exon 3 (primers RsmA-5 and -6). The products of the first two reactions were mixed together and a new PCR was performed with primers RsmA-1 and -4 to fuse exons 1 and 2. Finally, the product of the latter reaction was mixed with the product of the reaction of exon 3 and a PCR was performed with primers RsmA-1 and RsmA-6 to obtain the entire CDS, from the ATG to the last codon (next to the Stop codon but not including it). This fragment was digested with the enzymes EcoRI and XbaI and inserted in the pPICZ-B vector digested with the same enzymes, so that the* Pc-rsmA* gene ORF was fused in frame with the sequences in the vector encoding the *c-myc* epitope and the 6xHis tag. (C) For* Pc-atf21*, the previously cloned gene was used as template for amplification with primers AtfB-1 and AtfB-2, since this gene contains no introns. The cloning procedure in vector pPICZ-B was as described above. (Asterisks indicate additional amino acids in the fusion proteins resulting from the cloning strategies. In the case of* Pc-atf21*, the cloning strategy resulted in the loss of the last amino acid, Asn).

**Additional file 6**. Purification of recombinant* c-myc*-6xHis-tagged PcYap1, PcRsmA and PcAtf21 proteins after expression in* P. pastoris*. Protein extracts were obtained as described in Materials and Methods and loaded onto Ni-NTA Spin 50 (Qiagen) columns. The tagged proteins were eluted with increasing concentrations of imidazole. Aliquots of the eluted samples were analyzed by SDS-PAGE, lane M: size marker, lane CE: protein crude extract, imidazole concentrations were 100, 250, 300 and 500 mM for PcYap1::*c-myc*-6xHis, and 50, 200, 300 and 500 mM for PcRsmA::*c-myc*-6xHis and PcAtf21::*c-myc*-6xHis. Bottom right panel, partially purified PcRsmA::*c-myc*-6xHis and PcAtf21::*c-myc*-6xHis run on the same gel for size comparison purposes.

**Additional file 7**. EMSA to analyze possible interactions between PcYap1 and PcRsmA upon binding to their respective binding sites. Probes containing either the PcYap1-binding site (PTA1-WT), the PcRsmA binding site (RsmA-2C) or both (upPta1) were incubated with their respective binding proteins (lanes 2, 4, 6, 7) or with both proteins (lanes 3, 5, 8). The results show that no apparent interaction between the proteins occurs when one of them recognizes and binds its specific site (lanes 3, 5). In lane 8 a supershift takes place. Probe upPta1 contains both binding sites for each of the proteins, therefore the supershift pattern does not demonstrate interaction between the proteins and can be explained by the simultaneous binding of the proteins to their respective sites; nevertheless, interaction when this simultaneous binding occurs cannot be excluded.

**Additional file 8**. Clustal W alignment of PcYap1 with* S. cerevisiae* Yap1 and *S. pombe* Pap1, and conservation of functional domains between them. Nuclear import and export sequences (NLS and NES) are highly conserved. Also conserved are the cysteine-rich domains N-CRD and C-CRD, the latter overlapping the NES, and the key cysteine residues forming disulphide bonds described in Yap1 which mask the NES and prevent export form the nucleus, resulting in nuclear location of the protein in oxidative conditions (see text for details).

**Additional file 9**. Clustal W alignment of PcRsmA with* A. nidulans* RsmA,* C. albicans* FCR3, and* S. cerevisiae* Yap3. An NLS is well conserved in all proteins at the same position as in the Yap1 proteins (the beginning of the bZIP domain). Three putative NES were detected with LocNES (from Support Vector Machine) in the amino acid sequence of PcRsmA; two of them, NES-2 and NES-3, show some conservation with the equivalent regions in the other three proteins. NES-3 is located at the C-terminus and between the only two cysteine residues present in PcRsmA, which should form a disulphide bond in oxidative conditions that would cause the conformational change detected by SDS-PAGE (Fig. 7). A cysteine rich domain located at the N-terminus in Yap3 is not conserved in any of the other proteins.

**Additional file 10**. Radial growth of strains with knocked down expression of *Pc-yap1* and* Pc-rsmA* (A) and strains overexpressing the respective genes (B). Strain C1 contains the empty pGpdPki-RNAi vector used for gene silencing and strain C2 (*pyrG*) contains the empty pBKSpyrG vector used for gene overexpression. Three microliters of a suspension with 1x10^4^ conidia/mL were inoculated on the center of a Petri dish with PDA medium. The cultures were incubated in the dark at 28 °C for 144 h. Every 24 h the diameter of the colonies was measured.

**Additional file 11**. Conidia production of strains overexpressing *Pc-yap1* or* Pc-rsmA* in Power medium without added H_2_O_2_. Strain C2 (*pyrG*) contains the empty pBKSpyrG vector used for gene overexpression. Bar sizes are the result of three biological replicas, error bars correspond to standard deviation. See Materials and Methods for details.

**Additional file 12**. Oligonucleotides used in this work. Added restriction enzyme sites at the 5’ end of several primers are highlighted in color.

## Data Availability

All data generated or analyzed during this study are included in this published article and its additional files.
