# Supplementary figures and images for: bZIP transcription factors PcYap1 and PcRsmA link oxidative stress response to secondary metabolism and development in Penicillium chrysogenum
Source: Microb Cell Fact. 2022 Apr 2;21:50. doi: 10.1186/s12934-022-01765-w (PMC8977021; doi:10.1186/s12934-022-01765-w)

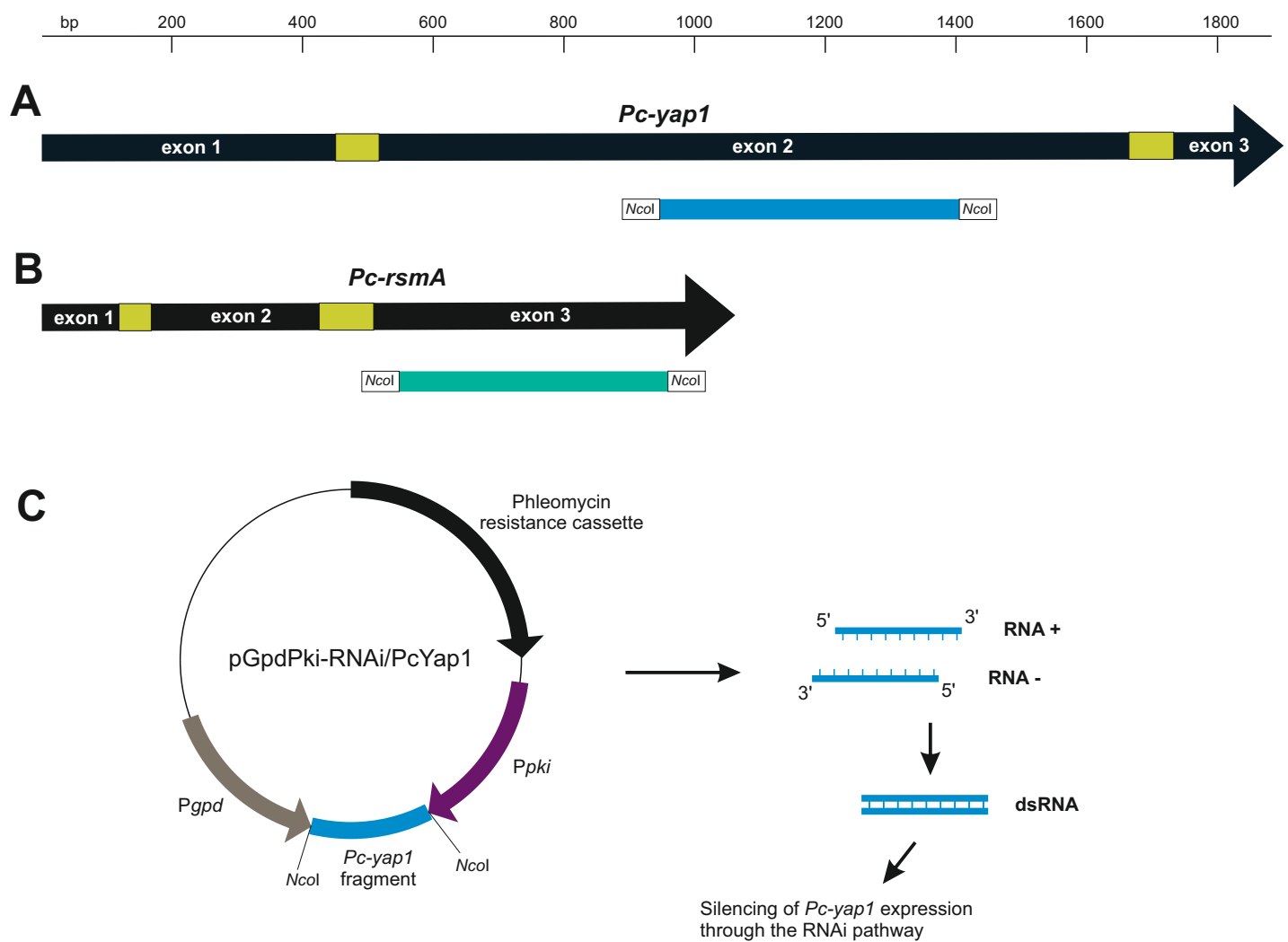

Supplement: Supplementary file 1 — Additional file 1. Strategy for silencing of expression of Pc-yap1 and Pc-rsmA. (A) A DNA fragment of 458 bp from the exon 2 of Pc-yap1 was amplified by PCR with primers siYAP1-F and -R, digested with NcoI and inserted at the NcoI site of plasmid pGpdPki-RNAi to obtain plasmid pGpdPki-RNAi/PcYap1. (B) A DNA fragment of 409 bp from the exon 3 of the Pc-rsmA gene was amplified by PCR with primers siRsmA-F and -R, digested with NcoI and inserted at the NcoI site of plasmid pGpdPki-RNAi to obtain plasmid pGpdPki-RNAi/PcRsmA. (C) Strategy of silencing. Transcription from the opposite-oriented promoters gpd and pki generates complementary RNA strands that form a dsRNA with the sequence of the inserted 458 bp fragment from Pc-yap1. This dsRNA will cause silencing of the expression of Pc-yap1 through the RNAi pathway. [file 12934_2022_1765_MOESM1_ESM.pdf]

A

pPyrG-*pki::Pc-yap1*

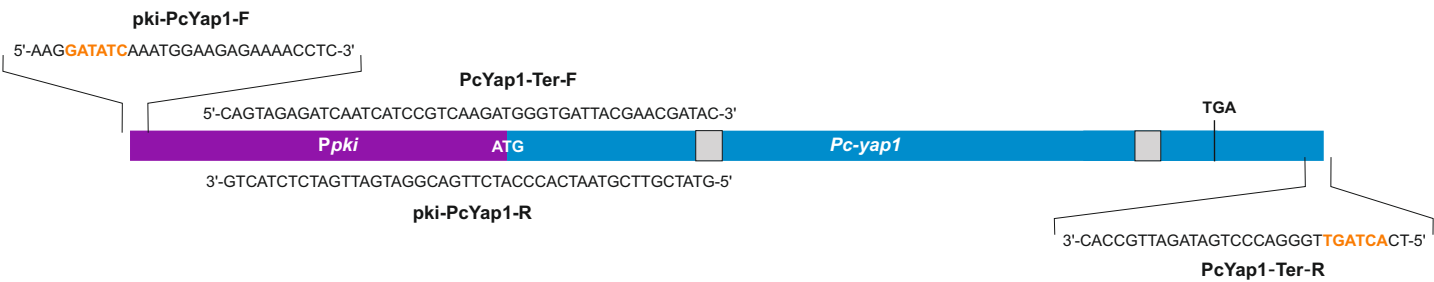

B

pPyrG-*pki::Pc-rsmA*

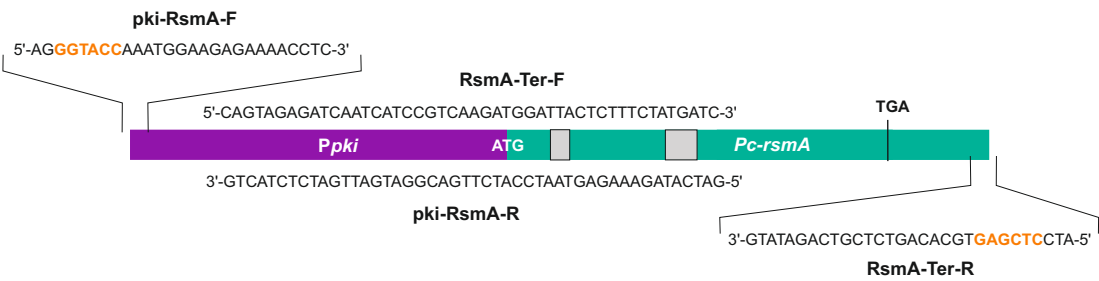

Supplement: Supplementary file 2 — Additional file 2. Recombinant PCR for overexpression of genes Pc-yap1 and Pc-rsmA. The pki gene promoter from A. niger was fused to fragments from the genes extending from the ATG start codon to around 300 bp downstream the TGA stop codon to ensure the presence of the transcriptional terminator. The position of the primers used for PCR reactions is indicated (see Materials and Methods for details). The final fragments with the genes fused to the pki promoter were digested with the restriction enzymes EcoRV and SpeI (Ppki::Pc-yap1) or KpnI and XhoI (Ppki::Pc-rsmA) and inserted in the vector pBKSpyrG to obtain the plasmids pPyrG-pki::Pc-yap1 and pPyrG-pki::Pc-rsmA, respectively; the restriction sites in the primers are highlighted in color. Introns are indicated in grey color. Added restriction sites at the 5’-end of the primers are highlighted in orange color. [file 12934_2022_1765_MOESM2_ESM.pdf]

# A

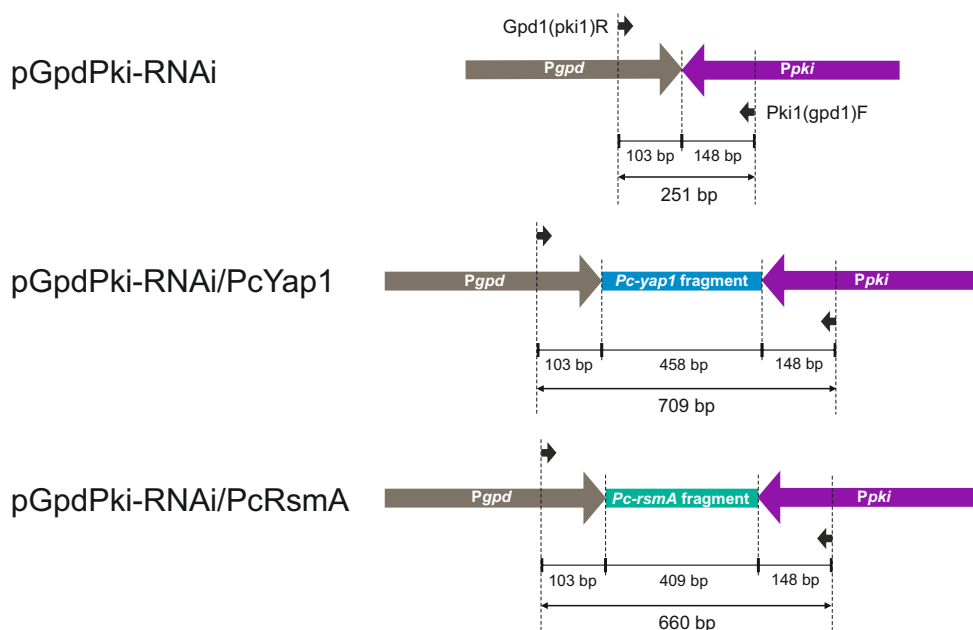

# B

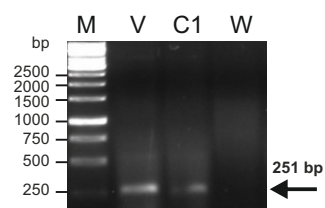

# C

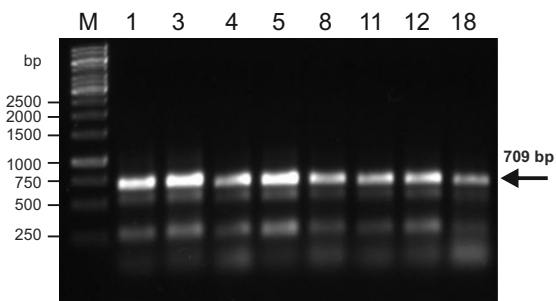

# D

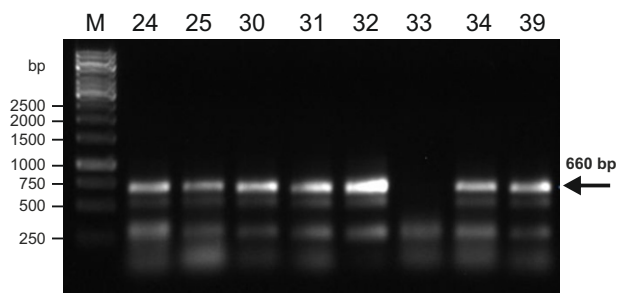

# E

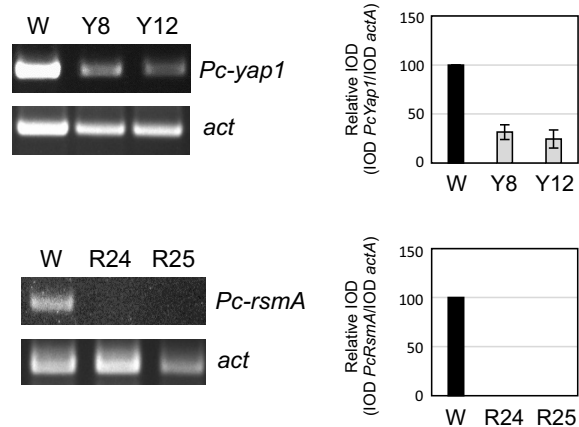

Supplement: Supplementary file 3 — Additional file 3. Confirmation of the presence of plasmids for RNAi-mediated silencing of Pc-yap1 and Pc-rsmA and analysis of silencing in P. chrysogenum transformants. (A) Close-up of the pGpdPki-RNAi vector region with the opposite-oriented gpd and pki promoters, and the plasmids pGpdPki-RNAi/PcYap1 and pGpdPki-RNAi/PcRsmA with the inserted DNA fragments from the Pc-yap1 and Pc-rsmA genes at the NcoI site. Primers Gpd1(pki1)R and Pki1(gpd1)F are shown at the position of annealing with sequences in the gpd and pki promoters, respectively. The expected size of amplified DNA fragments in each type of transformant is indicated with double-headed arrows. (B) Agarose gel with the result of PCR amplification with primers Gpd1(pki1)R and Pki1(gpd1)F using as template DNA from the purified pGpdPki-RNAi vector (lane V), DNA from strain C1 containing the pGpdPki-RNAi vector (lane C1) and DNA from strain Wis54-1255 (lane W). (C) Results of the PCR amplification performed with the mentioned primers and DNA from eight transformants with the pGpdPki-RNAi/PcYap1 plasmid (Y1…. Y18). (D) Results of the PCR amplification performed with the mentioned primers and DNA from eight transformants with the pGpdPki-RNAi/PcRsmA plasmid (R24… R39). (E) Silencing of expression of Pc-yap1 (upper panels) and Pc-rsmA (lower panels) in selected strains containing the RNAi-silencing plasmids pGpdPki-RNAi/PcYap1 and pGpdPki-RNAi/PcRsmA, respectively. RNA was extracted from mycelium grown for 60 h in MPPY medium and used for semiquantitative RT-PCR (as described by Domínguez-Santos et al. [96]) using primers qPcYap1-F and -R for analysis of Pc-yap1 expression, qRsmA-F and -R for Pc-rsmA, and qactA-F and -R for act. The left panels show the intensity of the bands in an agarose gel loaded with the products of the RT-PCR reactions, and the right panels the densitometry analysis of the bands. The results were normalized with the bands of the constitutively expressed act gene. The parental strain Wis54-12 [file 12934_2022_1765_MOESM3_ESM.pdf]

pPICZ-A/PcYap1

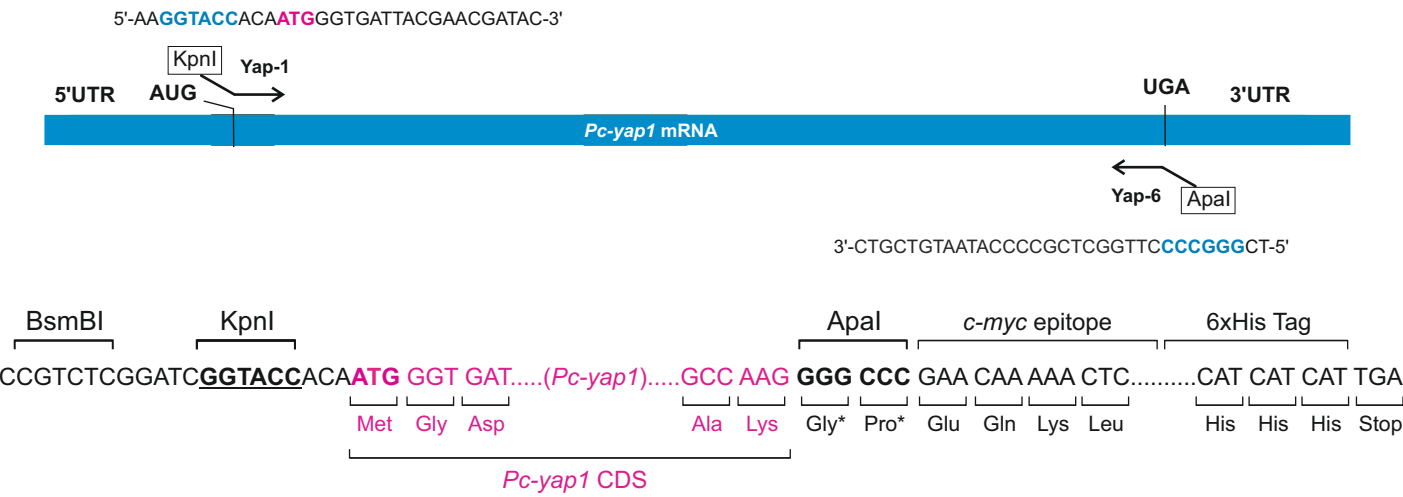

pPICZ-B/PcRsmA

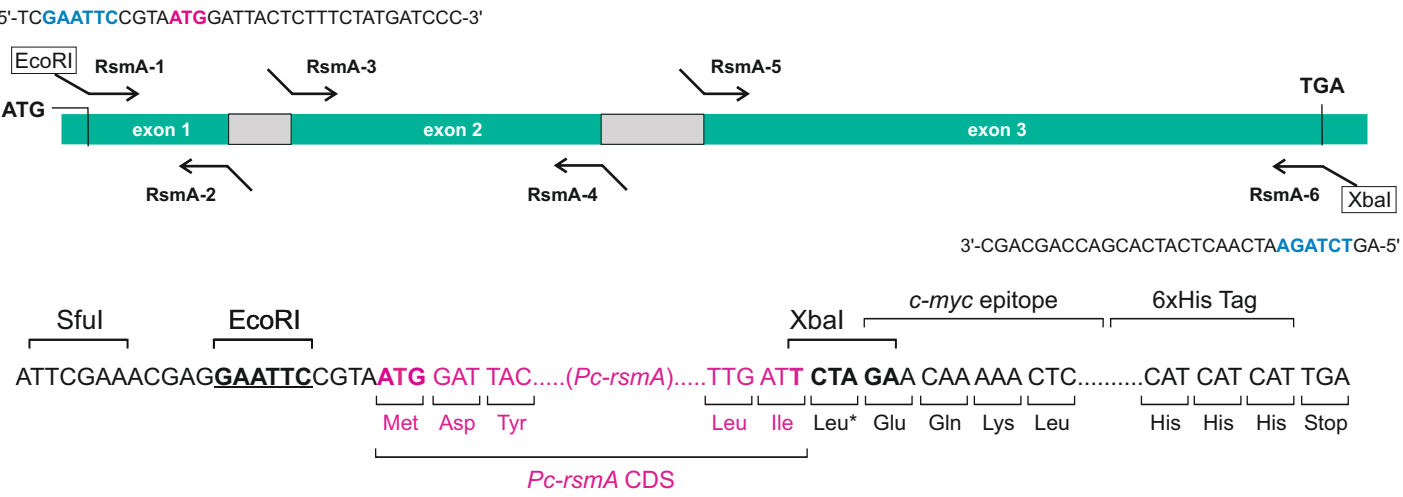

pPICZ-B/PcAtf21

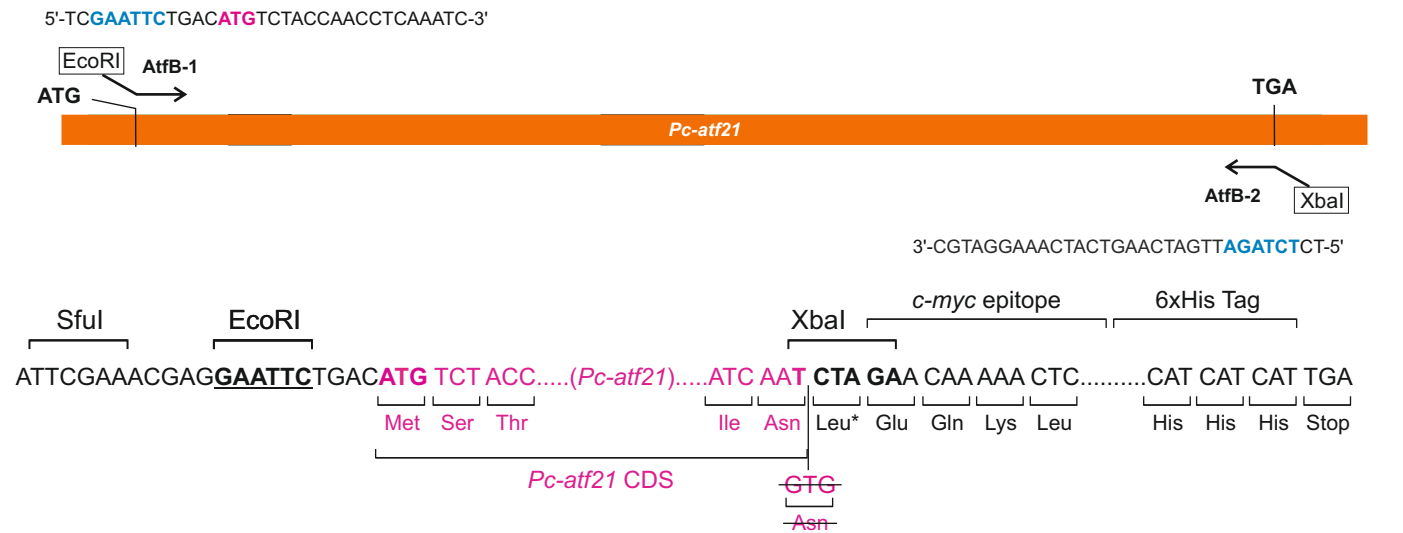

Supplement: Supplementary file 5 — Additional file 5. Isolation of Pc-yap1, Pc-rsmA and Pc-atf21 CDS and insertion in expression vectors for P. pastoris. (A) An RT-PCR was performed using RNA from a submerged culture and primers Yap-1 and Yap-6, which contain restriction sites for KpnI and ApaI, respectively. The amplified fragment was inserted in the pPICZ-A vector after digestion with these enzymes and ligation. The in-frame insertion is shown, indicating the position of the cloning enzymes, the Pc-yap1 CDS and the c-myc-6xHis tag. (B) For Pc-rsmA, a recombinant PCR approach was followed, designing primers with sequences linking exon 1 to 2 (RsmA-2 and RsmA-3) and exon 2 to 3 (RsmA-4 and RsmA-5), plus primer RsmA-1, annealing at the start of the ORF and containing an EcoRI restriction site at 5’, and primer RsmA-6, annealing at the end of the ORF and containing an XbaI restriction site. PCR reactions were performed to separately amplify exon 1 (primers RsmA-1 and -2), exon 2 (primers RsmA-3 and -4) and exon 3 (primers RsmA-5 and -6). The products of the first two reactions were mixed together and a new PCR was performed with primers RsmA-1 and -4 to fuse exons 1 and 2. Finally, the product of the latter reaction was mixed with the product of the reaction of exon 3 and a PCR was performed with primers RsmA-1 and RsmA-6 to obtain the entire CDS, from the ATG to the last codon (next to the Stop codon but not including it). This fragment was digested with the enzymes EcoRI and XbaI and inserted in the pPICZ-B vector digested with the same enzymes, so that the Pc-rsmA gene ORF was fused in frame with the sequences in the vector encoding the c-myc epitope and the 6xHis tag. (C) For Pc-atf21, the previously cloned gene was used as template for amplification with primers AtfB-1 and AtfB-2, since this gene contains no introns. The cloning procedure in vector pPICZ-B was as described above. (Asterisks indicate additional amino acids in the fusion proteins resulting from the cloning strategies. In the case of [file 12934_2022_1765_MOESM5_ESM.pdf]

**A**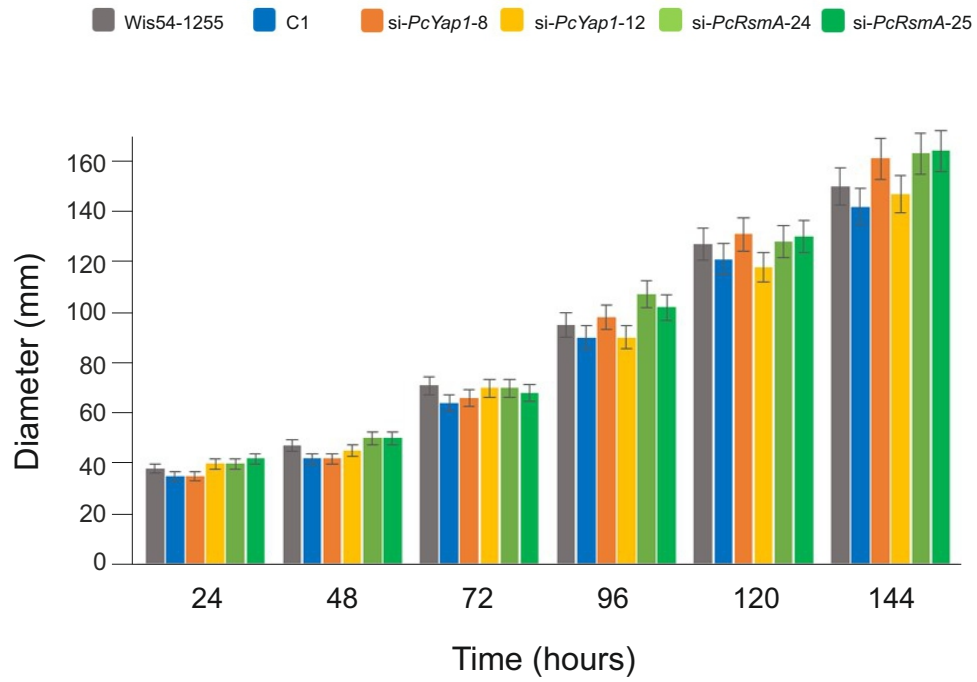**B**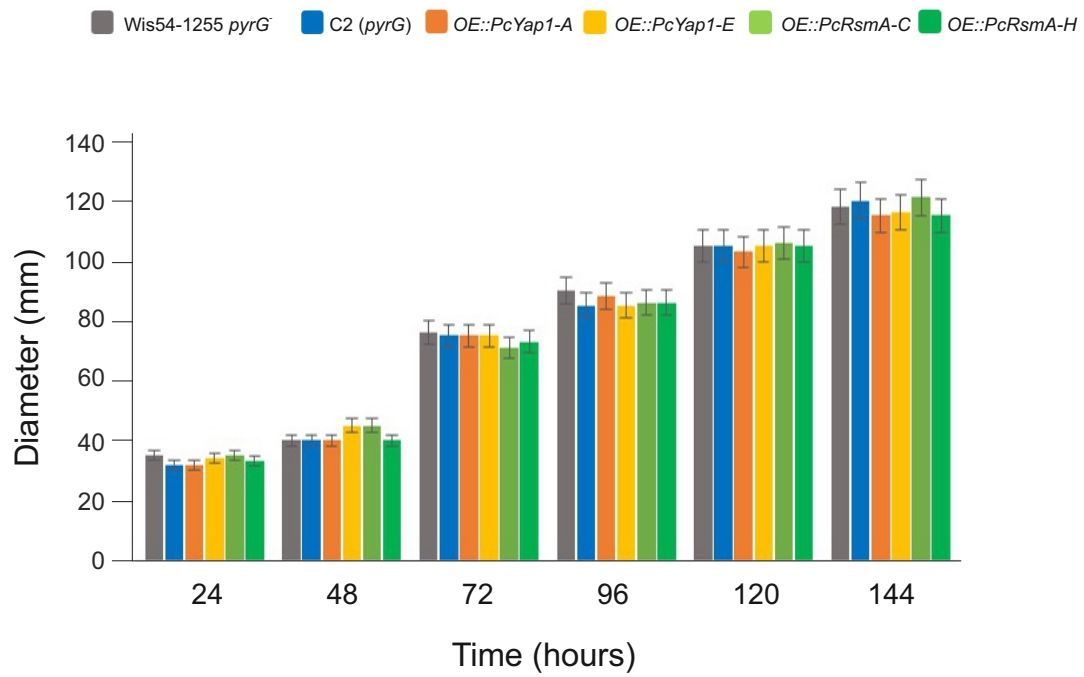

Supplement: Supplementary file 10 — Additional file 10. Radial growth of strains with knocked down expression of Pc-yap1 and Pc-rsmA (A) and strains overexpressing the respective genes (B). Strain C1 contains the empty pGpdPki-RNAi vector used for gene silencing and strain C2 (pyrG) contains the empty pBKSpyrG vector used for gene overexpression. Three microliters of a suspension with 1x10^4^ conidia/mL were inoculated on the center of a Petri dish with PDA medium. The cultures were incubated in the dark at 28 °C for 144 h. Every 24 h the diameter of the colonies was measured. [file 12934_2022_1765_MOESM10_ESM.pdf]

Wis54-1255 *pyrG* C2 (*pyrG*) *OE::PcYap1-A* *OE::PcYap1-E* *OE::PcRsmA-C* *OE::PcRsmA-H*

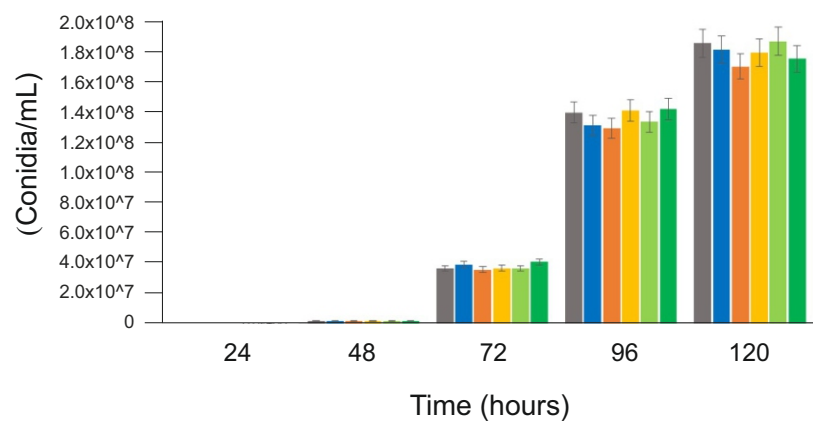

Supplement: Supplementary file 11 — Additional file 11. Conidia production of strains overexpressing Pc-yap1 or Pc-rsmA in Power medium without added H2O2. Strain C2 (pyrG) contains the empty pBKSpyrG vector used for gene overexpression. Bar sizes are the result of three biological replicas, error bars correspond to standard deviation. See Materials and Methods for details. [file 12934_2022_1765_MOESM11_ESM.pdf]
