# Supplementary material for: bZIP transcription factors PcYap1 and PcRsmA link oxidative stress response to secondary metabolism and development in Penicillium chrysogenum
Source: Microb Cell Fact. 2022 Apr 2;21:50. doi: 10.1186/s12934-022-01765-w (PMC8977021; doi:10.1186/s12934-022-01765-w)
Supplement: Supplementary file 4 — Additional file 4. Confirmation of the presence of plasmids for overexpression of Pc-yap1 and Pc-rsmA in P. chrysogenum transformants. (A) Scheme of the fusion of the pki promoter with the Pc-yap1 (top) and Pc-rsmA (down) genes at the ATG start codon in the plasmids pPyrG-pki::Pc-yap1 and pPyrG-pki::Pc-rsmA. Primers Pki1(gpd1)F, OE-PcYap1-R, siYAP1-R, OE-RsmA-R and siRSMA-R are shown at the position of annealing with sequences in the pki promoter and the Pc-yap1 and Pc-rsmA genes, respectively. The expected size of amplified DNA fragments in each type of transformant for every primer pair is indicated with double-headed arrows. (B) Agarose gel with the result of PCR amplification with primers Pki1(gpd1)F and OE-PcYap1-R using as template DNA from the purified pPyrG-pki::Pc-yap1 plasmid (lane P), DNA from strain Wis54-1255 (lane W), a transformant with the empty pBKSpyrG vector (lane Y) and transformants OE::PcYap1-A through –F (lanes A through F). Transformants selected for further characterization are indicated with a red circle. (C) Result of PCR amplification with primers Pki1(gpd1)F and OE-RsmA-R using as template DNA from transformants OE::PcRsmA-A through –H (lanes A through H). (D) Result of PCR amplification with primers Pki1(gpd1)F and siRSMA-R using as template DNA from the purified pPyrG-pki::Pc-rsmA plasmid (lane P), total DNA from strain Wis54-1255 (lane W), a transformant with the empty pBKSpyrG vector (lane Y) and transformants OE::Pc-rsmA-C through –H (lanes C through H). Transformants selected for further characterization are indicated with a red circle. [file 12934_2022_1765_MOESM4_ESM.pdf]

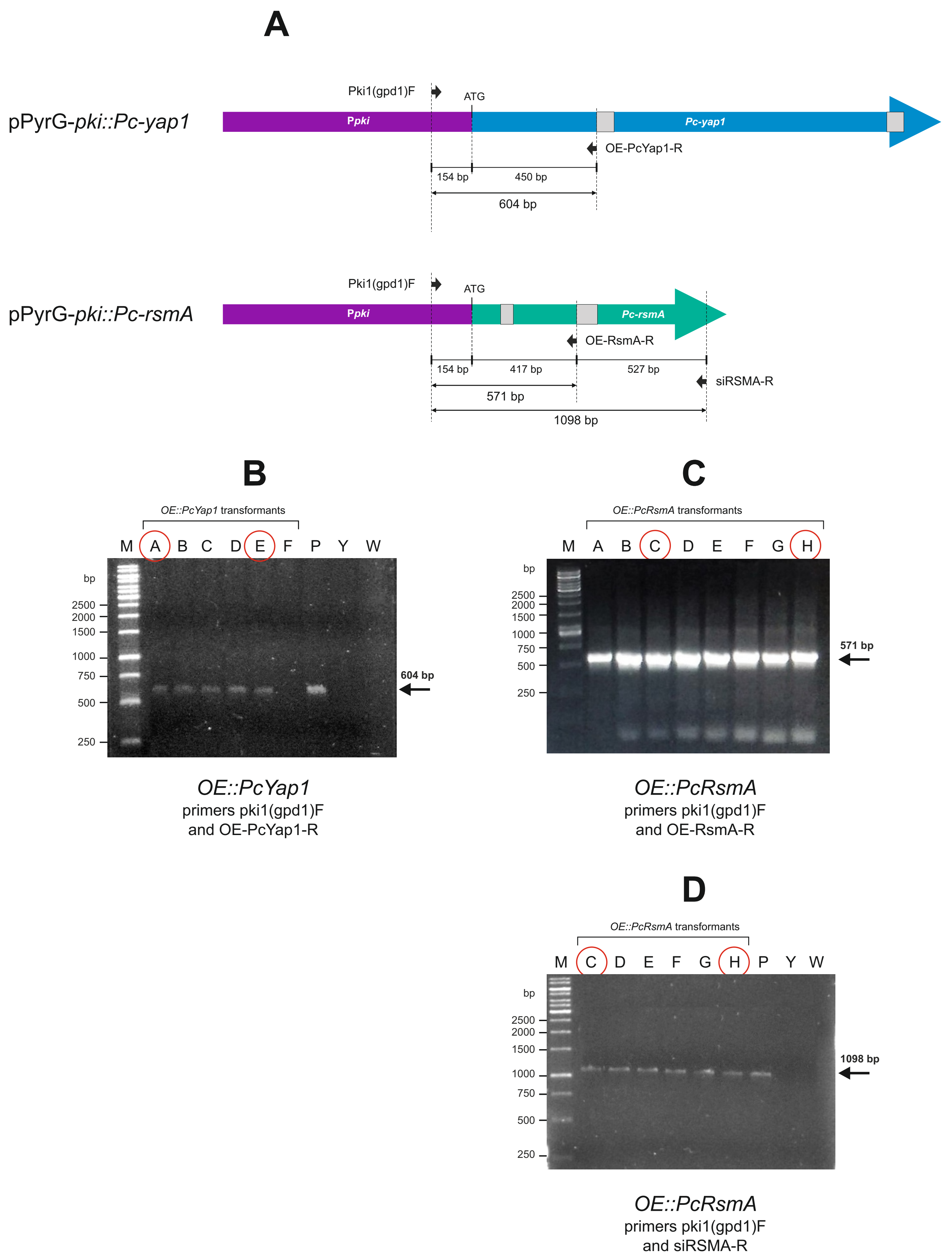

A
pPyrG-pki::Pc-yap1
Pki1(gpd1)F
ATG
Ppki
Pc-yap1
OE-PcYap1-R
154 bp
450 bp
604 bp
pPyrG-pki::Pc-rsmA
Pki1(gpd1)F
ATG
Ppki
Pc-rsmA
OE-RsmA-R
154 bp
417 bp
527 bp
siRSMA-R
571 bp
1098 bp
B
OE::PcYap1 transformants
M A B C D E F P Y W
bp
2500
2000
1500
1000
750
500
250
604 bp
OE::PcYap1
primers pki1(gpd1)F
and OE-PcYap1-R
C
OE::PcRsmA transformants
M A B C D E F G H
bp
2500
2000
1500
1000
750
500
250
571 bp
OE::PcRsmA
primers pki1(gpd1)F
and OE-RsmA-R
D
OE::PcRsmA transformants
M C D E F G H P Y W
bp
2500
2000
1500
1000
750
500
250
1098 bp
OE::PcRsmA
primers pki1(gpd1)F
and siRSMA-R
