# Supplementary material for: bZIP transcription factors PcYap1 and PcRsmA link oxidative stress response to secondary metabolism and development in Penicillium chrysogenum
Source: Microb Cell Fact. 2022 Apr 2;21:50. doi: 10.1186/s12934-022-01765-w (PMC8977021; doi:10.1186/s12934-022-01765-w)
Supplement: Supplementary file 6 — Additional file 6. Purification of recombinant c-myc-6xHis-tagged PcYap1, PcRsmA and PcAtf21 proteins after expression in P. pastoris. Protein extracts were obtained as described in Materials and Methods and loaded onto Ni-NTA Spin 50 (Qiagen) columns. The tagged proteins were eluted with increasing concentrations of imidazole. Aliquots of the eluted samples were analyzed by SDS-PAGE, lane M: size marker, lane CE: protein crude extract, imidazole concentrations were 100, 250, 300 and 500 mM for PcYap1::c-myc-6xHis, and 50, 200, 300 and 500 mM for PcRsmA::c-myc-6xHis and PcAtf21::c-myc-6xHis. Bottom right panel, partially purified PcRsmA::c-myc-6xHis and PcAtf21::c-myc-6xHis run on the same gel for size comparison purposes. [file 12934_2022_1765_MOESM6_ESM.pdf]

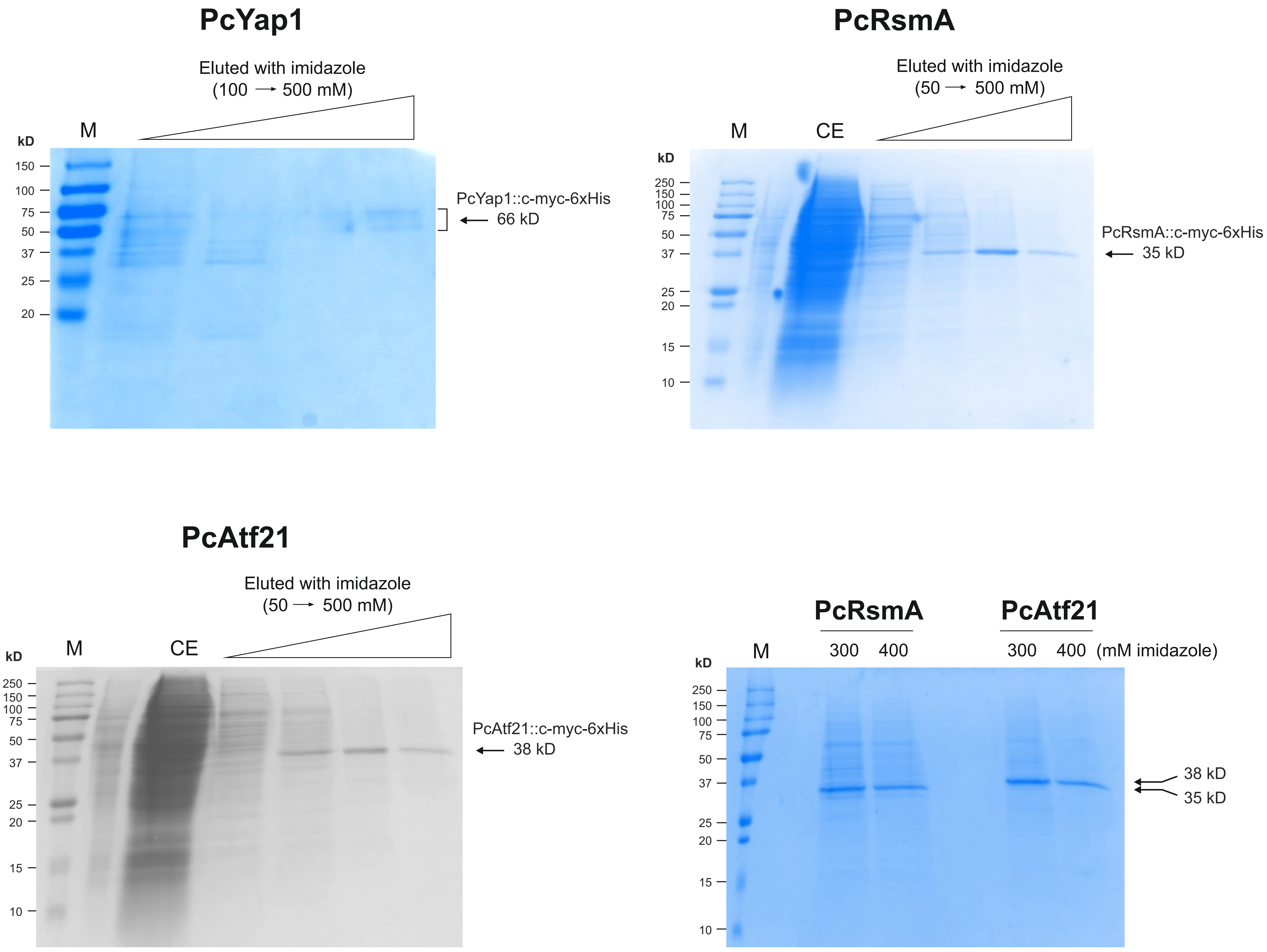

PcYap1
Eluted with imidazole
(100 → 500 mM)
M
kD
150
100
75
50
37
25
20
PcYap1::c-myc-6xHis
66 kD
PcRsmA
Eluted with imidazole
(50 → 500 mM)
M
CE
kD
250
150
100
75
50
37
25
20
15
10
PcRsmA::c-myc-6xHis
35 kD
PcAtf21
Eluted with imidazole
(50 → 500 mM)
M
CE
kD
250
150
100
75
50
37
25
20
15
10
PcAtf21::c-myc-6xHis
38 kD
PcRsmA
PcAtf21
M
300
400
300
400
(mM imidazole)
kD
250
150
100
75
50
37
25
20
15
10
38 kD
35 kD
