# Supplementary material for: bZIP transcription factors PcYap1 and PcRsmA link oxidative stress response to secondary metabolism and development in Penicillium chrysogenum
Source: Microb Cell Fact. 2022 Apr 2;21:50. doi: 10.1186/s12934-022-01765-w (PMC8977021; doi:10.1186/s12934-022-01765-w)
Supplement: Supplementary file 7 — Additional file 7. EMSA to analyze possible interactions between PcYap1 and PcRsmA upon binding to their respective binding sites. Probes containing either the PcYap1-binding site (PTA1-WT), the PcRsmA binding site (RsmA-2C) or both (upPta1) were incubated with their respective binding proteins (lanes 2, 4, 6, 7) or with both proteins (lanes 3, 5, 8). The results show that no apparent interaction between the proteins occurs when one of them recognizes and binds its specific site (lanes 3, 5). In lane 8 a supershift takes place. Probe upPta1 contains both binding sites for each of the proteins, therefore the supershift pattern does not demonstrate interaction between the proteins and can be explained by the simultaneous binding of the proteins to their respective sites; nevertheless, interaction when this simultaneous binding occurs cannot be excluded. [file 12934_2022_1765_MOESM7_ESM.pdf]

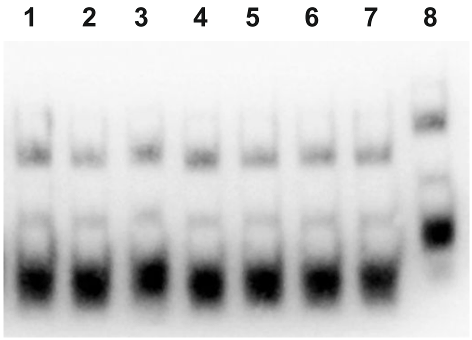

	Probe	Protein/s
**1.**	RsmA-2	PcRsmA
**2.**	PTA1-WT	PcYap1
**3.**	PTA1-WT	PcYap1 + PcRsmA
**4.**	RsmA-2C	PcRsmA
**5.**	RsmA-2C	PcYap1 + PcRsmA
**6.**	upPta1	PcYap1
**7.**	upPta1	PcRsmA
**8.**	upPta1	PcYap1 + PcRsmA
