# Supplementary material for: bZIP transcription factors PcYap1 and PcRsmA link oxidative stress response to secondary metabolism and development in Penicillium chrysogenum
Source: Microb Cell Fact. 2022 Apr 2;21:50. doi: 10.1186/s12934-022-01765-w (PMC8977021; doi:10.1186/s12934-022-01765-w)
Supplement: Supplementary file 8 — Additional file 8. Clustal W alignment of PcYap1 with S. cerevisiae Yap1 and S. pombe Pap1, and conservation of functional domains between them. Nuclear import and export sequences (NLS and NES) are highly conserved. Also conserved are the cysteine-rich domains N-CRD and C-CRD, the latter overlapping the NES, and the key cysteine residues forming disulphide bonds described in Yap1 which mask the NES and prevent export form the nucleus, resulting in nuclear location of the protein in oxidative conditions (see text for details). [file 12934_2022_1765_MOESM8_ESM.pdf]

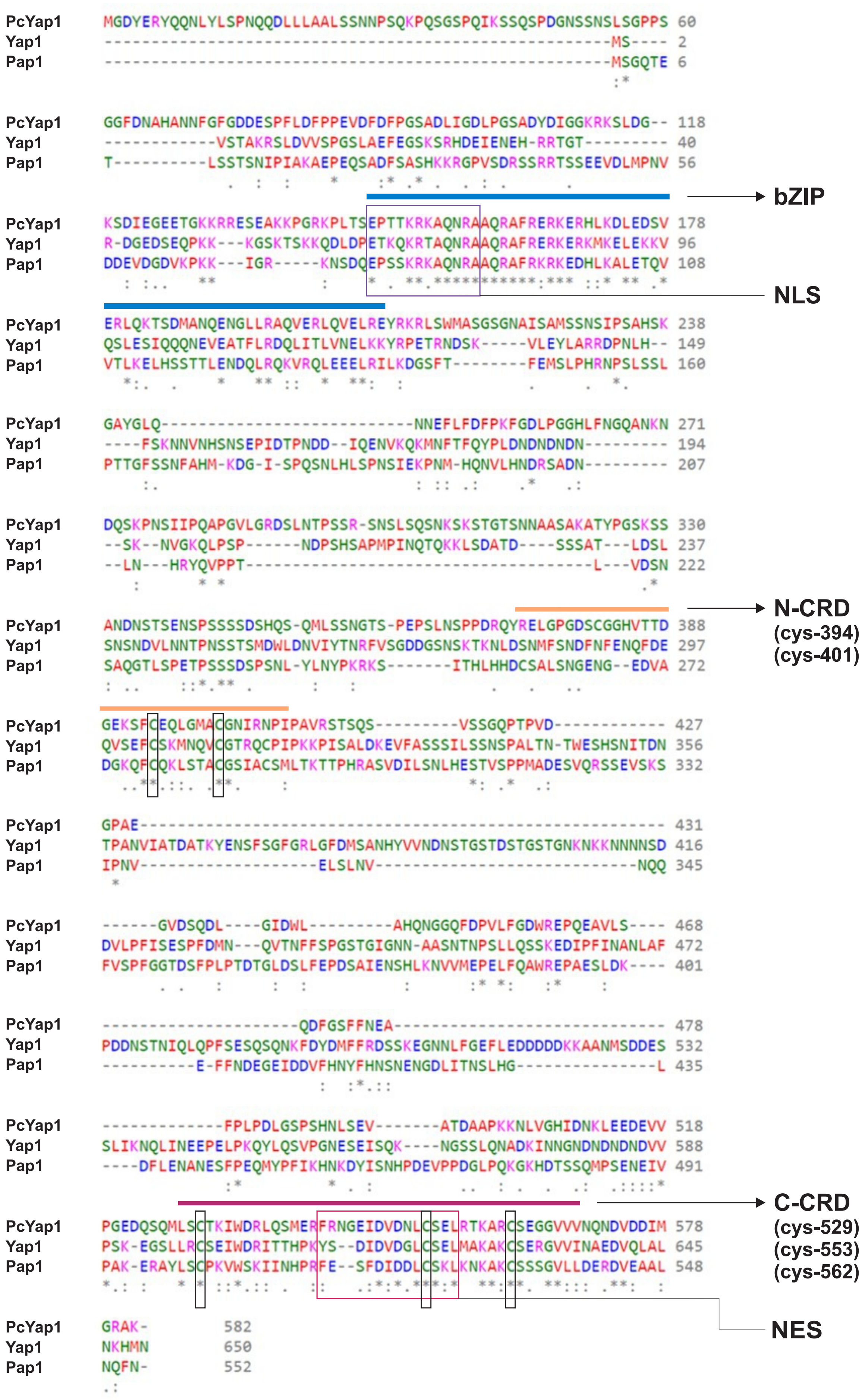
PcYap1
Yap1
Pap1
bZIP
NLS
N-CRD
(cys-394)
(cys-401)
C-CRD
(cys-529)
(cys-553)
(cys-562)
NES
