# Supplementary material for: bZIP transcription factors PcYap1 and PcRsmA link oxidative stress response to secondary metabolism and development in Penicillium chrysogenum
Source: Microb Cell Fact. 2022 Apr 2;21:50. doi: 10.1186/s12934-022-01765-w (PMC8977021; doi:10.1186/s12934-022-01765-w)
Supplement: Supplementary file 9 — Additional file 9. Clustal W alignment of PcRsmA with A. nidulans RsmA, C. albicans FCR3, and S. cerevisiae Yap3. An NLS is well conserved in all proteins at the same position as in the Yap1 proteins (the beginning of the bZIP domain). Three putative NES were detected with LocNES (from Support Vector Machine) in the amino acid sequence of PcRsmA; two of them, NES-2 and NES-3, show some conservation with the equivalent regions in the other three proteins. NES-3 is located at the C-terminus and between the only two cysteine residues present in PcRsmA, which should form a disulphide bond in oxidative conditions that would cause the conformational change detected by SDS-PAGE (Fig. 7). A cysteine rich domain located at the N-terminus in Yap3 is not conserved in any of the other proteins. [file 12934_2022_1765_MOESM9_ESM.pdf]

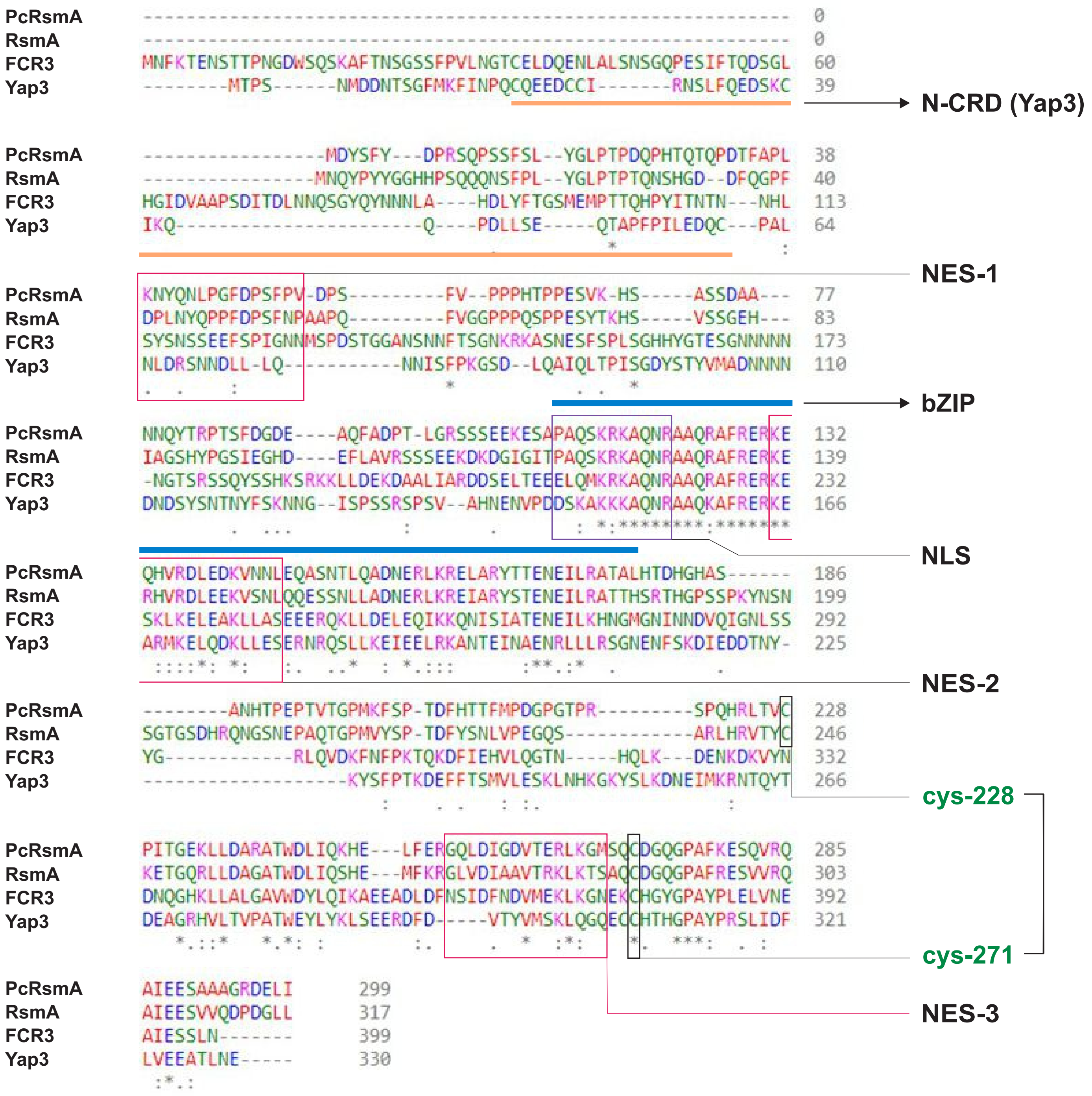
PcRsmA
RsmA
FCR3
Yap3
MNFKTENSTTPNGDWSQSKAFTNSGSSFPVLNGTCELDQENLALSNSGQPESIFTQDSGL 60
--------MTPS------NMDDNTSGFMKFINPQCQEEDCCI-------RNSLFQEDSKC 39
N-CRD (Yap3)
----------------MDYSFY---DPRSQPSSFSL--YGLPTPDQPHTQTQPDTFAPL 38
---------------MNQYPYYGGHHPSQQQNSFPL--YGLPTPTQNSHGD--DFQGPF 40
HGIDVAAPSDITDLNNQSGYQYNNNLA----HDLYFTGSMEMPTTQHPYITNTN---NHL 113
IKQ---------------------Q----PDLLSE-----QTAPFPILEDQC---PAL 64
NES-1
KNYQNLPGFDPSFPV-DPS---------FV--PPPHTPPESVK-HS-----ASSDAA--- 77
DPLNYQPPFDPSFNPAAPQ---------FVGGPPPQSPPESYTKHS-----VSSGEH--- 83
SYSNSSEEFSPIGNNMSPDSTGGANSNNFTSGNKRKASNESFSPLSGHHYGTESGNNNNN 173
NLDRSNNDLL-LQ------------NNISFPKGSD--LQAIQLTPISGDYSTYVMADNNNN 110
bZIP
NNQYTRPTSFDGDE----AQFADPT-LGRSSSEEKESAPAQSKRKAQNRAAQRAFRERKE 132
IAGSHYPGSIEGHD----EFLAVRSSSEEKDKDGIGITPAQSKRKAQNRAAQRAFRERKE 139
-NGTSRSSQYSSHKSRKKLLDEKDAALIARDDSELTEEELQMKRKAQNRAAQRAFRERKE 232
DNDSYSNTNYFSKNNG--ISPSSRSPSV--AHNENVPDDSKAKKKAQNRAAQKAFRERKE 166
NLS
QHVRDLEDKVNNLEQASNTLQADNERLKRELARYTTENEILRATALHTDHGHAS------ 186
RHVRDLEEKVSNLQQESSNLLADNERLKREIARYSTENEILRATTHSRTHGPSSPKYNSN 199
SKLKELEAKLLASEEERQKLLDELEQIKKQNISIATENEILKHNGMGNINNDVQIGNLSS 292
ARMKELQDKLLESERNRQSLLKEIEELRKANTEINAENRLLLRSGNENFSKDIEDDTNY- 225
NES-2
--------ANHTPEPTVTGPMKFSP-TDFHTTFMPDGPGTPR---------SPQHRLTVC 228
SGTGSDHRQNGSNEPAQTGPMVYSP-TDFYSNLVPEGQS------------ARLHRVTYC 246
YG-----------RLQVDKFNFPKTQKDFIEHVLQGTN-----HQLK---DENKDKVYN 332
------------------KYSFPTKDEFFTSMVLESKLNHKGKYSLKDNEIMKRNTQYT 266
cys-228
PITGEKLLDARATWDLIQKHE---LFERGQLDIGDVTERLKGMSQCDGQGPAFKESQVRQ 285
KETGQRLLDAGATWDLIQSHE---MFKRGLVDIAAVTRKLKTSAQCDGQGPAFRESVVRQ 303
DNQGHKLLALGAVWDYLQIKAEEADLDFNSIDFNDVMEKLKGNEKCHGYGPAYPLELVNE 392
DEAGRHVLTVPATWEYLYKLSEERDFD-----VTYVMSKLQGQECCHTHGPAYPRSLIDF 321
cys-271
AIEESAAAGRDELI 299
AIEESVVQDPDGLL 317
AIESSLN------- 399
LVEEATLNE----- 330
NES-3
