# Supplementary material for: bZIP transcription factors PcYap1 and PcRsmA link oxidative stress response to secondary metabolism and development in Penicillium chrysogenum
Source: Microb Cell Fact. 2022 Apr 2;21:50. doi: 10.1186/s12934-022-01765-w (PMC8977021; doi:10.1186/s12934-022-01765-w)
Supplement: Supplementary file 12 — Additional file 12. Oligonucleotides used in this work. Added restriction enzyme sites at the 5’ end of several primers are highlighted in color. [file 12934_2022_1765_MOESM12_ESM.pdf]

Oligo name	Sequence (5'→3')	Purpose
pcbAB-pcbC-1F	ATCGGCGTGGTTCCGTTC	PCR for EMSA probe 1
pcbAB-pcbC-1R	ATGGAGATTGGCCAGGTCAG	PCR for EMSA probe 1
pcbAB-pcbC-2F	CTGGCCAATCTCCATCG	PCR for EMSA probe 2
pcbAB-pcbC-2R	TTGCCCACTGCCAAGTC	PCR for EMSA probe 2
pcbAB-pcbC-3F	CTTGGCAGTGGGCAAAC	PCR for EMSA probe 3
pcbAB-pcbC-3R	AACGACGGCGATGTTCC	PCR for EMSA probe 3
pcbAB-pcbC-4F	AACATCGCCGTCGTTGAG	PCR for EMSA probe 4
pcbAB-pcbC-4R	TCAGGCCAATCGTTGATG	PCR for EMSA probe 4
pcbAB-pcbC-5F	CCGAGCATCAACGATTGG	PCR for EMSA probe 5
pcbAB-pcbC-5R	TGTTTATGCTGAGACAACCTG	PCR for EMSA probe 5
pcbAB-pcbC-6F	ATGTCTCCAGGTTGTCTC	PCR for EMSA probe 6
pcbAB-pcbC-6R	GGAAGCCATGGTGTCTAG	PCR for EMSA probe 6
AflR-RsmA-F	CGACTGACACAAGAAATAACAATTC	EMSA probe AflR-RsmA
AflR-RsmA-R	GAATTGTTATTTCTTGTGTCAGTCG	EMSA probe AflR-RsmA
upPta1-RsmA-F	GAGATACGGTTACTAATT	PCR for EMSA probe upPta1
upPta1-RsmA-R	TGTTTATGCTGAGACAACCT	PCR for EMSA probe upPta1
dwPta1-F	CCGAGCATCAACGATTGGC	PCR for EMSA probe dwPta1
dwPta1-R	CCGTATCTCGAAGTCGGAG	PCR for EMSA probe dwPta1
RsmA-2-F	CAGTAAGCATCTGGGCTGCAAGCGTATAATGTCTCCAGGTTGTCTCAGCATAAACA	EMSA probe RsmA-2
RsmA-2-R	TGTTTATGCTGAGACAACCTGGAGACATTATACGCTTGCAGCCCAGATGCTTACTG	EMSA probe RsmA-2
RsmA-2A-F	CAGTAAGCATCTGGGCTGCAAGCGTATA	EMSA probe RsmA-2A
RsmA-2A-R	TATACGCTTGCAGCCCAGATGCTTACTG	EMSA probe RsmA-2A
RsmA-2B-F	CAGTAAGCATCTGGGCTGCAAGCGTATAATGTCTCCAG	EMSA probe RsmA-2B
RsmA-2B-R	CTGGAGACATTATACGCTTGCAGCCCAGATGCTTACTG	EMSA probe RsmA-2B
RsmA-2C-F	CTCCAGGTTGTCTCAGCATAAACA	EMSA probe RsmA-2C
RsmA-2C-R	TGTTTATGCTGAGACAACCTGGAG	EMSA probe RsmA-2C
PTA1-WT-F	GCCAGTGTAAAATTAGTAACCGTATCTC	EMSA probe PTA1-WT
PTA1-WT-R	GAGATACGGTTACTAATTTTACACTGGC	EMSA probe PTA1-WT
PTA1-M1-F	GCCAGTGTAAAACTAGTCACCGTATCTC	EMSA probe PTA1-M1
PTA1-M1-R	GAGATACGGTGACTAGTTTTACACTGGC	EMSA probe PTA1-M1
NorR_4-F	TTTCAACATTTCTTGAGTACTTTTCTAAGCCGTGACATAATGAACGGATCA	EMSA probe NorR4
NorR_4-R	TGATCCGTTCATTATGTCACGGCTTAGAAAAGTACTCAAGAAATGTTGAAA	EMSA probe NorR4
penDE-CRE123-F	AGGTCCTGCATTAGCGTACGCCCCAGGTTTAGATCAAAGTTACGTAATTTATATGTA	EMSA probe penDE-CRE123
penDE-CRE123-R	TACATATAAATTACGTAACTTTGATCTAAACCTGGGGCGTACGCTAATGCAGGACCT	EMSA probe penDE-CRE123
penDE-CRE123-M1-F	AGGTCCTGCATTAGCGTACGCCCCAGGTTTAGATCAAAGCTACGAAATTTATATGTA	EMSA probe penDE-CRE123-M1
penDE-CRE123-M1-R	TACATATAAATTTCGTAGCTTTGATCTAAACCTGGGGCGTACGCTAATGCAGGACCT	EMSA probe penDE-CRE123-M1
AP1-brlA-F	AAAACAATACAATTTACTAAAGTCCACTGGGCA	EMSA probe AP1-brlA
AP1-brlA-R	TGCCCAGTGGACTTTAGTAAATTGTATTGTTTT	EMSA probe AP1-brlA
AP1-brlA-MutF	AAAACAATACAATTGACTAGAGTCCACTGGGCA	EMSA probe AP1-brlA-Mut
AP1-brlA-MutR	TGCCCAGTGGACTCTAGTCAATTGTATTGTTTT	EMSA probe AP1-brlA-Mut
Yap1-F	TAGGATCCATAGACCTGCGATAAGCACACCC	Cloning of *Pc-yap1*
Yap1-R	AATCTAGACCCTTACAGCTTTCTTCTGGAGT	Cloning of *Pc-yap1*
RsmA-F	ACGGATCCTATGACGTTTCGGATTCTGTACC	Cloning of *Pc-rsmA*
RsmA-R	CATAAGCTTGACGAGGACGACTACAAGAAAGA	Cloning of *Pc-rsmA*
AtfB-F	TCGGATCCATCTGTTCGGTATTTGGGCTACT	Cloning of *Pc-atfB*
AtfB-R	AGAATTCGGAGTTTCTCAACCCCGTATATC	Cloning of *Pc-atfB*
RsmA-1	TCGAATTCCGTAATGGATTACTCTTTCTATGATCCC	Insertion into pPICZ-B
RsmA-2	AGCCAGGGAGGTTTTGGTAATTCTTCAAAGGGGCGAATGTATCTGGTT	Intron splicing in *Pc-rsmA*
RsmA-3	AACCAGATACATTCGCCCCTTTGAAGAATTACCAAAACCTCCCTGGCT	Intron splicing in *Pc-rsmA*
RsmA-4	CTCCCGGAACGCTCGTTGAGCTGCTCGGTTCTGTGCCTT	Intron splicing in *Pc-rsmA*
RsmA-5	AAGGCACAGAACCGAGCAGCTCAACGAGCGTTCCGGGAG	Intron splicing in *Pc-rsmA*
RsmA-6	AGTCTAGAATCAACTCATCACGACCAGCAGC	Insertion into pPICZ-B
Yap-1	AAGGTACCACAATGGGTGATTACGAACGATAC	Insertion into pPICZ-A
Yap-6	TCGGGCCCCTTGGCTCGCCCCATAATGTCGTC	Insertion into pPICZ-A
AtfB-1	TCGAATTCTGACATGTCTACCAACCTCAAATC	Insertion into pPICZ-B
AtfB-2	TCTCTAGATTGATCAAGTCATCAAAGGATGC	Insertion into pPICZ-B
siYAP1-F	TAACCATGGACTCCTTCGTCTCGATCCAACTC	Insertion into pGpdPki-RNAi
siYAP1-R	TACCCATGGTGGTGAGCCAACCAATCAATCC	Insertion into pGpdPki-RNAi
siRSMA-F	TAACCATGGCGCGACCTGGAAGACAAAGTAAA	Insertion into pGpdPki-RNAi
siRSMA-R	TACCCATGGACACTGCGACATACCCTTCAAAC	Insertion into pGpdPki-RNAi

Pki1(gpd1)F	TCTCTCTTCTCTCGCTCAC	Confirm RNAi transformants
Gpd1(pki1)R	TACTCCATCCTTCCCATCC	Confirm RNAi transformants
qPcYap1-F	ACAATGCCCATGCCAACAATTTC	qRT-PCR for *Pc-yap1*
qPcYap1-R	GAAGAGATGACCACCAGGCAAATC	qRT-PCR for *Pc-yap1*
qRsmA-F	TTCGCTATACGGGCTGCCTAC	qRT-PCR for *Pc-rsmA*
qRsmA-R	CATCGGGCATGAAGGTGGTAT	qRT-PCR for *Pc-rsmA*
qactA-F	AGAAGTTGCTGCTCTCGTCA	qRT-PCR for *act*
qactA-R	CGACAATGGAAGGGAAAACA	qRT-PCR for *act*
pki-PcYap1-F	AAGGATATCAAATGGAAGAGAAAACCTC	*OE::PcYap1*
PcYap1-Ter-F	CAGTAGAGATCAATCATCCGTCAAGATGGGTGATTACGAACGATAC	*OE::PcYap1*
pki-PcYap1-R	GTATCGTTCGTAATCACCCATCTTGACGGATGATTGATCTCTACTG	*OE::PcYap1*
PcYap1-Ter-R	TCACTAGTTGGGACCCTGATAGATTGCCAC	*OE::PcYap1*
pki-RsmA-F	AGGGTACCAAATGGAAGAGAAAACCTC	*OE::PcRsmA*
RsmA-Ter-F	CAGTAGAGATCAATCATCCGTCAAGATGGATTACTCTTTCTATGATC	*OE::PcRsmA*
pki-RsmA-R	GATCATAGAAAGAGTAATCCATCTTGACGGATGATTGATCTCTACTG	*OE::PcRsmA*
RsmA-Ter-R	ATCCTCGAGTGCACAGTCTCGTCAGATATG	*OE::PcRsmA*
OE-PcYap1-R	CGTAGTAGGCTCTGAAGTCAGTGG	Confirm *OE::PcYap1* transf.
OE-RsmA-R	GCTGCTCGGTTCTGTGCCTT	Confirm *OE::PcRsmA* transf.
N-brlA-1F	TCTTCCTGGATACGACGAGAATGT	PCR for Northern probe *brlA*
N-brlA-1R	AGGAGCTGTTTCTTGGAGGATTTG	PCR for Northern probe *brlA*
N-actA-1F	CACCACGAGCTGTTTTCCCTT	PCR for Northern probe *act*
N-actA-1R	TCAGCGAGGATCTTCATCAGGTAGT	PCR for Northern probe *act*
